# Nationwide Surveillance on Antimicrobial Resistance Profiles of *Enterococcus faecium* and *Enterococcus faecalis* Isolated from Healthy Food Animals in South Korea, 2010 to 2019

**DOI:** 10.3390/microorganisms9050925

**Published:** 2021-04-26

**Authors:** Mi Hyun Kim, Dong Chan Moon, Su-Jeong Kim, Abraham Fikru Mechesso, Hyun-Ju Song, Hee Young Kang, Ji-Hyun Choi, Soon-Seek Yoon, Suk-Kyung Lim

**Affiliations:** Bacterial Disease Division, Animal and Plant Quarantine Agency, 177 Hyeksin 8-ro, Gimcheon-si 39660, Gyeongsangbuk-do, Korea; kimmh940301@naver.com (M.H.K.); ansehdcks@korea.kr (D.C.M.); kimsujeong27@gmail.com (S.-J.K.); abrahamf@korea.kr (A.F.M.); shj0211@korea.kr (H.-J.S.); kanghy7734@korea.kr (H.Y.K.); wlgus01@korea.kr (J.-H.C.); yoonss24@korea.kr (S.-S.Y.)

**Keywords:** antimicrobial resistance, *E. faecium*, *E. faecalis*, food animals, public health

## Abstract

Intestinal commensal bacteria are considered good indicators for monitoring antimicrobial resistance. We investigated the antimicrobial resistance profiles and resistance trends of *Enterococcus faecium* and *Enterococcus faecalis* isolated from food animals in Korea between 2010 and 2019. *E. faecium* and *E. faecalis,* isolated from chickens and pigs, respectively, presented a relatively high resistance rate to most of the tested antimicrobials. We observed high ciprofloxacin (67.9%), tetracycline (61.7%), erythromycin (59.5%), and tylosin (53.0%) resistance in *E. faecium* isolated from chickens. Similarly, more than half of the *E. faecalis* isolates from pigs and chickens were resistant to erythromycin, tetracycline and tylosin. Notably, we observed ampicillin, daptomycin, tigecycline and linezolid resistance in a relatively small proportion of enterococcal isolates. Additionally, the enterococcal strains exhibited an increasing but fluctuating resistance trend (*p* < 0.05) to some of the tested antimicrobials including daptomycin and/or linezolid. *E. faecalis* showed higher Multidrug resistance (MDR) rates than *E. faecium* in cattle (19.7% vs. 8.6%, respectively) and pigs (63.6% vs. 15.6%, respectively), whereas a comparable MDR rate (≈60.0%) was noted in *E. faecium* and *E. faecalis* isolated from chickens. Collectively, the presence of antimicrobial-resistant *Enterococcus* in food animals poses a potential risk to public health.

## 1. Introduction

Enterococci are commensal bacteria of the gastrointestinal tract of animals and humans. They can also be detected in the environment and in foods of animal origin. They are considered emerging pathogens of humans and are often associated with invasive nosocomial infections [1]. Enterococci have emerged as good indicators of antibiotic resistance. They can acquire resistance genes from other bacteria, which can also spread to other commensal and pathogenic bacteria through horizontal transfer of mobile genetic elements [2]. Besides, the frequent use of antimicrobials in humans and animals select for resistant enterococci [3].

There are 55 enterococci species reported so far based on *16S rDNA* sequences. *Enterococcus faecium* and *Enterococcus faecalis* are the most commonly isolated species, accounting for more than 80% of isolates [4]. *E. faecium* and *E. faecalis* have become increasingly important pathogens worldwide because they are associated with life-threatening hospital-acquired infections, whereas the remaining *Enterococcus* spp. are infrequent causes of human clinical infections [3]. *E. faecalis* is the most pathogenic species, while *E. faecium* is commonly involved in the acquisition and transfer of antimicrobial resistance [5].

Food animals have been suggested as a possible intermediate vector in the transmission mode of antimicrobial-resistant enterococci. Cross-contamination of edible carcass tissues during the slaughter process represents a significant food safety hazard [6]. Tyson et al. [7] and Boehm et al. [8] demonstrated that more than 90% of food samples from animals are contaminated with *Enterococcus* at the slaughterhouse, predominantly with *E. faecium* and *E. faecalis.* Therefore, it is important to continuously evaluate the antimicrobial resistance profiles of *E. faecium* and *E. faecalis* strains found in food animals.

Monitoring the resistance profiles and temporal trends of antimicrobial resistance in *Enterococcus* species isolated from food animals provides useful information for understanding the level of resistance of gut microbial flora as well as for the empirical selection of antimicrobial agents to treat infected patients. Several researchers have reported the antimicrobial resistance profiles of *Enterococcus* isolated from food animals worldwide [9,10,11,12,13,14]. In South Korea (Korea), only a few studies have been conducted to determine the resistance profiles of *E. faecium* and *E. faecalis* isolates from food animals [15,16,17,18,19,20]. These studies were conducted in isolates collected from some parts of the country for a very short duration, and hence the resistance trends of the *Enterococcus* isolates remained unexplored. Thus, we performed this study to determine the antimicrobial resistance profiles and resistance trends of *E. faecium* and *E. faecalis* isolated from healthy cattle, pigs and chickens throughout Korea between 2010 and 2019.

## 2. Materials and Methods

### 2.1. Bacterial Isolates

A total of 3360 *E. faecium* isolates (572 isolates from cattle, 1385 from pigs, and 1403 from chickens) and 4218 *E. faecalis* isolates (910 isolates from cattle, 1556 from pigs, and 1752 from chickens) were obtained from 16 laboratories/centers participating in the Korean Veterinary Antimicrobial Resistance Monitoring System between 2010 and 2019. The isolates were recovered from the feces of animals in various slaughterhouses (Supplementary Table S1). No more than five fecal samples were collected from each farm. Sample processing and enterococcal isolation were performed according to Lim et al. [20] using buffered peptone water and *Enterococcus* agar media (Becton, Dickinson, Sparks, MD). *Enterococcus* species were identified by Polymerase Chain Reaction (PCR) [21] or matrix-assisted laser desorption ionization-time-of-flight mass spectrometry (MALDI-TOF, Biomerieux, Marcy L’Etoile, France). One isolate per sample was selected for the antimicrobial susceptibility test.

### 2.2. Antimicrobial Susceptibility Study

The antimicrobial susceptibility profiles of the enterococcal isolates were determined by the broth microdilution method [22], using commercially available antibiotic-containing plates (Sensititre, Trek Diagnostics, Cleveland, OH, USA). The following antimicrobials were tested: ampicillin (1–16 µg/mL), chloramphenicol (2–32 µg/mL), ciprofloxacin (0.25–16 µg/mL), daptomycin (0.5–32 µg/mL), erythromycin (1–64 µg/mL), florfenicol (2–32 µg/mL), gentamicin (128–2048 µg/mL), kanamycin (128–2048 µg/mL), linezolid (0.5–16 µg/mL), quinupristin/dalfopristin (1–32 µg/mL), salinomycin (2–32 µg/mL), streptomycin (128–2048 µg/mL), tetracycline (2–128 µg/mL), tigecycline (0.12–2 µg/mL), tylosin (1–64 µg/mL) and vancomycin (2–32 µg/mL). *Enterococcus faecalis* ATCC 29212 was used as a quality reference strain. The MIC values were interpreted according to the guidelines of the Clinical and Laboratory Standards Institute [22], the National Antimicrobial Resistance Monitoring System [23], and the Danish Integrated Antimicrobial Resistance Monitoring and Research Programme [24]. MIC50 and MIC90 values were defined as the lowest concentration of antimicrobials at which 50% and 90% of the isolates were inhibited, respectively. Isolates that were resistant to at least three subclasses of antimicrobials, excluding resistance to quinupristin/dalfopristin in *E. faecalis*, were considered multidrug-resistant.

### 2.3. Data Analysis

Analysis of the antimicrobial resistance rates and Pearson correlation was conducted using Excel (Microsoft-Excel, 2016, Microsoft Corporation, Redmond, WA, USA) and Rex software (Version 3.0.3, RexSoft Inc., Seoul, Korea). *p* values less than 0.05 were considered significant.

## 3. Results

### 3.1. Antimicrobial Resistance

In general, a more frequent occurrence of resistance to most of the tested antimicrobials was observed among the *E. faecalis* isolates compared with *E. faecium* (Table 1). More than 50% of the *E. faecalis* isolates were resistant to macrolides and tetracycline, whereas we noted a moderate resistance rate to these antimicrobials in *E. faecium*. Both enterococcal species demonstrated a very low resistance rate (≤10.0%) to ampicillin, daptomycin, tigecycline, linezolid and salinomycin. However, all isolates were susceptible to vancomycin.

*E. faecium* isolates recovered from chickens demonstrated a very high resistance rate to most of the tested antimicrobials compared with those isolated from cattle and pigs (Table 1). More than half of the *E. faecium* isolates from chickens were resistant to ciprofloxacin, erythromycin, tetracycline, and tylosin. *E. faecium* isolates from cattle presented relatively low resistance rates (<10.0%) to the tested antimicrobials except to tetracycline (25.9%), ciprofloxacin (23.1%), and erythromycin (19.9%). Similarly, pig isolates exhibited low resistance rates (<15.0%) to most of the tested antimicrobials, except to erythromycin (31.5%) and tetracycline (20.1%). We observed linezolid (0.0–5.2%), daptomycin (5.6–11.1%), and tigecycline (5.5–6.8%) resistance in a relatively small percentage of *E. faecium* isolates. Indeed, the linezolid resistance rate was high in chicken isolates (5.2%) compared with those of cattle (0.3%) and pigs (0.0%). Additionally, we identified high-level gentamicin (1.3%, MIC ≥ 500 µg/mL), kanamycin (15.0%, MIC ≥ 500 µg/mL), and streptomycin (17.1%, MIC ≥ 2000 µg/mL) resistance in *E. faecium* isolated mainly from chickens and pigs (Supplementary Table S2). The MIC_50_ and MIC_90_ of the tested antimicrobials against *E. faecium* isolated from cattle, chickens, and pigs are summarized in Supplementary Table S3A–C.

*E. faecalis* isolated from chickens and pigs exhibited high resistance rate to most of the tested antimicrobials, especially to tetracycline (76.1–78.3%), erythromycin (63.0–67.1%), and tylosin (63.6–66.6%) (Table 1). In contrast, cattle isolates presented a moderate or low resistance rate to these antimicrobials. We identified linezolid (1%), daptomycin (0.8%), and tigecycline (10.2%) resistance in a very small proportion of *E. faecalis* isolates. High-level gentamicin (14%), kanamycin (28.5%), and streptomycin (34.2%) resistances were noted in *E. faecalis* isolated predominantly from chickens and pigs (Supplementary Table S4). The MIC_50_ and MIC_90_ of the tested antimicrobials against *E. faecalis* isolated from cattle, chickens, and pigs are summarized in Supplementary Table S5A–C.

### 3.2. Antimicrobial Resistance Trends

**Figure 1:**
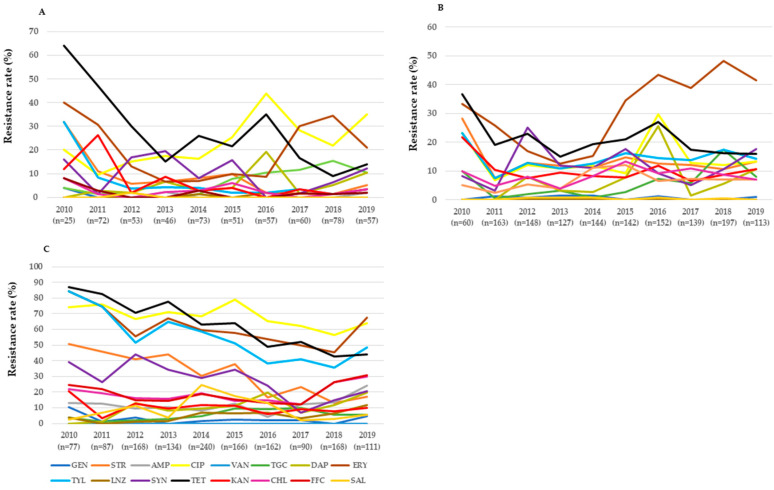
Antimicrobial resistance trend of *E. faecium* isolates recovered from cattle (**A**), pigs (**B**), and chickens (**C**) in Korea from 2010 to 2019. Abbreviations: AMP, ampicillin; CIP, ciprofloxacin; CHL, chloramphenicol; DAP, daptomycin; ERY, erythromycin; FFC, florfenicol; GEN, gentamicin; KAN, kanamycin; LIN, linezolid; SAL, salinomycin; STR, streptomycin; SYN, quinupristin/dalfopristin; TET, tetracycline; TGC tigecycline, TYL, tylosin; and VAN, vancomycin.

We observed variations in the antimicrobial resistance trend between the two strains as well as in the same strain from different sources. *E. faecium* isolates exhibited an increasing but fluctuating trend of resistance to ciprofloxacin and tigecycline (isolates from cattle), erythromycin (from pigs), and daptomycin and linezolid (from chickens) (Figure 1 and Supplementary Tables S6–S8). In contrast, we noted a decreasing but fluctuating resistance trend to some of the tested antimicrobials in *E. faecium* isolated from cattle (tetracycline, streptomycin, and tylosin) and chickens (streptomycin, ciprofloxacin, tetracycline, erythromycin, tylosin, and quinupristin/dalfopristin). Although we did not find a significant change in the daptomycin resistance trend in *E. faecium* isolated from cattle and pigs, resistance rates peaked in 2016.

**Figure 2:**
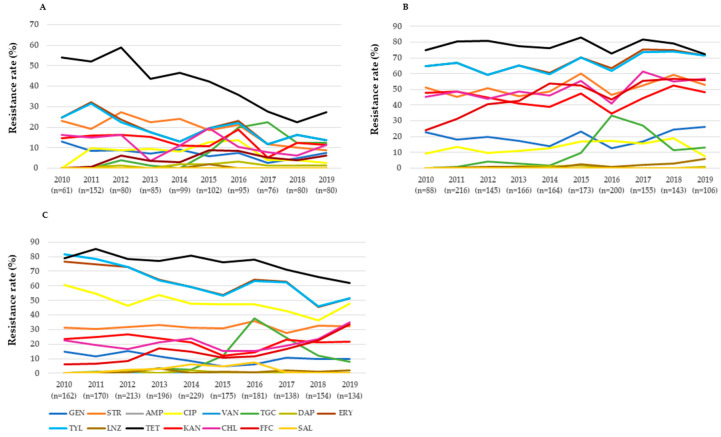
Antimicrobial resistance trend of *E. faecalis* isolates recovered from cattle (**A**), pigs (**B**), and chickens (**C**) in Korea from 2010 to 2019. Abbreviations: AMP, ampicillin; CIP, ciprofloxacin; CHL, chloramphenicol; DAP, daptomycin; ERY, erythromycin; FFC, florfenicol; GEN, gentamicin; KAN, kanamycin; LIN, linezolid; SAL, salinomycin; STR, streptomycin; TET, tetracycline; TGC, tigecycline, TYL, tylosin; and VAN, vancomycin.

*E. faecalis* isolates presented an increasing but fluctuating resistance trend to tigecycline (isolates from cattle), erythromycin, tigecycline, linezolid, and florfenicol (from pigs), and florfenicol (from chickens) (Figure 2 and Supplementary Tables S9–S11). Nevertheless, a decreasing but fluctuating resistance trend was noted in *E. faecalis* isolated from cattle (gentamicin, streptomycin, erythromycin, tylosin and tetracycline) and chickens (ciprofloxacin, erythromycin, tylosin and tetracycline). In addition, we noted a stable daptomycin resistance rate in *E. faecalis* isolated from cattle, chickens and pigs.

### 3.3. Multidrug Resistance (MDR) and Antimicrobial Resistance Patterns

The majority of *E. faecium* (72.0%) and *E. faecalis* (80.7%) isolates were resistant to one or more of the tested antimicrobials (Table 2 and Table 3). Notably, 33.3% of the *E. faecium* and 52.5% of the *E. faecalis* isolates were resistant to multiple antimicrobials (Table 1). MDR was high in *E. faecium* isolated from chickens (60.9%), and in *E. faecalis* recovered from pigs (63.6%) and chickens (59.7%). Besides, resistance to five or more antimicrobials was noted in *E. faecium* isolated from chickens (44.7%), and in *E. faecalis* from pigs (56.6%) and chickens (34.7%) (Table 2 and Table 3). A decreasing (*p* < 0.05) but fluctuating MDR trend was found in *E. faecium* and *E. faecalis* isolated from cattle and chickens, whereas the converse was noted in *E. faecalis* from pigs (Supplementary Tables S6, S8–S10 and S11).

A total of 434 and 262 MDR combination patterns were observed in *E. faecium* and *E. faecalis* isolates, respectively. Ciprofloxacin resistance with (5.2%) or without (5.7%) tetracycline was frequently noted in *E. faecium* isolated from chickens, whereas resistance to erythromycin was predominant in cattle (11%) and pig (12.9%) isolates (Table 2). Tetracycline resistance was frequently noted in *E. faecalis* isolated from cattle (14.5%) (Table 3). Tetracycline resistance with (11.0%) or without (11.5%) ciprofloxacin and macrolides were most frequent in *E. faecalis* isolated from chickens. The most frequent MDR pattern in *E. faecalis* isolated from pigs was resistance to seven antimicrobials (9.4%), including macrolides (Table 3).

## 4. Discussion

Knowledge of the distribution of antimicrobial-resistant bacteria in food animals and the food chain is vital for determining the potential risk to human health. Korea relies on testing isolates from food animals and foods of animal origin to determine the development and trends of antimicrobial resistance in the food chain. Consequently, this study provides a better understanding of the antimicrobial resistance profiles of the two most common enterococcal isolates recovered from healthy food animals slaughtered in Korea.

In this study, a considerable proportion of enterococcal isolates exhibited resistance to tetracycline and ciprofloxacin. Consistent with this study, high tetracycline resistance was reported in *E. faecium* and *E. faecalis* isolated from chickens and pigs in Korea [15,16,18,19,25], other Asian countries [13,26], Europe [27,28,29], and North America [30,31]. The ciprofloxacin resistance rate in *E. faecium* (67.9%) and *E. faecalis* (48.6%) isolated from chickens were higher than previous reports in Korea [15,16,18,25] but contradict reports from other countries [27,28,30,31,32,33]. About 85–100 tons of tetracyclines and 35–40 tons of quinolones (mainly enrofloxacin for poultry) were sold annually for the livestock industry in Korea during the study period [34]. Thus, the widespread use of these antimicrobials in livestock production provides selective pressure and accelerates the emergence of resistant *Enterococcus* strains.

The chloramphenicol and florfenicol resistance in *E. faecium* isolated from chickens (18.7–18.8%) and *E. faecalis* isolated from pigs (45.8–50.0%) were higher than those described in previous studies in Korea [15,18,25]. We also observed an increasing florfenicol resistance trend in *E. faecalis* isolated from pigs and chickens. Several studies in Asia and Europe reported variable chloramphenicol resistance rates in *E. faecium* (8–53%) and *E. faecalis* (16–53%) isolated from cattle, pigs and chickens [26,32,35,36]. The average annual consumption of florfenicol in Korean livestock especially in chickens and pigs was increased by about 50% in the last five years compared to the amount during 2010–2014 [34]. Thus, the frequent use of florfenicol in food animals might select for chloramphenicol and florfenicol resistance. Our recent studies also identified the oxazolidinone and phenicol resistance genes (*fexA*, *optrA*, and *poxtA*) in *Enterococcus* strains recovered from food animals and their carcasses in Korea. The *optrA* and *poxtA* genes were transferred to recipient strains from about 48% and 28% of *optrA* and *poxtA*-carrying enterococcal strains, respectively. Further, the *fexA* gene was co-transferred with the *optrA* gene in all *optrA-*positive transconjugants [17,37,38]. Therefore, horizontal dissemination of phenicol resistance genes among enterococcal isolates might also contribute to the increase in chloramphenicol and florfenicol resistance.

*Enterococcus* strains identified in this study demonstrated a relatively low resistance rate to the tested aminoglycosides; except to kanamycin and streptomycin in *E. faecalis* isolated from pigs. Additionally, high-level gentamicin, kanamycin and streptomycin resistance were noted among the *Enterococcus* strains. Previous studies in Asian countries [13,18,25,26] and Europe [27,29,36] have reported highly variable gentamicin (8–95%), kanamycin (38–62%), and streptomycin (62–100%) resistance rates in *E. faecium* and *E. faecalis* isolated from various food animals. Although enterococci are intrinsically resistant to clinically achievable concentrations of aminoglycosides, they are considered the antimicrobials of choice to treat human enterococcal infections when combined with cell wall inhibitors [4]. The widespread application of aminoglycosides in food animals (50, 71, and 296 tons in cattle, chickens and pigs, respectively, between 2010 and 2019) in Korea could be associated with the emergence of resistance [34]. Of note, the emergence of high-level aminoglycoside-resistant strains cannot be ignored because it could threaten the existing efficacy of the broad-spectrum activity of aminoglycosides.

Resistance to penicillin may narrow the therapeutic options for enterococcal infections [39]. Consistent with previous reports in Korea [18,25] and other countries [10,13,31,35,40], 4.8% of *E. faecium* isolates, especially those isolated from chickens, and 0.1% of *E. faecalis* were resistant to ampicillin. In contrast, previous studies have reported a relatively high ampicillin resistance in *E. faecium* from poultry in Germany (28%) [27], and *E. faecalis* from pigs in Thailand (44%) and Laos (12%) [26]. Ampicillin and penicillin are the most active β-lactams against *Enterococcus* inhibiting the synthesis of peptidoglycan [41]. Although we do not have information about the resistance determinants, intrinsic tolerance to the action of β-lactamase in *E. faecium* is associated with the presence of a species-specific chromosomal gene, *pbp5*, which encodes a class B penicillin-binding protein (PBP) with low affinity for ampicillin. Maintenance of peptidoglycan in the stationary phase mediated by an l,d-transpeptidase (Ld_tfm_) and overproduction of β-lactamase have also been implicated in ampicillin resistance in *E. faecium* [41]. Similarly, the overproduction of β-lactamase and point mutations of penicillin-binding protein (PBP4) have been shown to confer ampicillin resistance in *E. faecalis* [41,42].

Macrolide-lincosamide-streptogramin antibiotics constitute an alternative therapy for the treatment of insidious enterococcal infections [43]. We noted high erythromycin and tylosin resistance in *E. faecium* isolated from chickens and *E. faecalis* from pigs and chickens. Worryingly, despite fluctuations, we found an increasing erythromycin resistance trend in *E. faecium* and *E. faecalis* obtained from pigs. Consistent with this study, a considerable proportion of *E. faecium* and *E. faecalis* isolates obtained from food animals, especially chickens and pigs, in Korea demonstrated resistance to erythromycin (31–90%) and tylosin (50–94%) [16,18,19]. Our findings were also consistent with previous studies in Asia [26,40,44], Europe, and the United States [28,29,31,32,33]. Recently, the Korea Animal Health Products Association reported an increase in the annual sales of macrolides, especially tylosin, for livestock uses. The extensive use of macrolides (209 tons in pigs between 2010 and 2019), especially tylosin in Korean livestock husbandry can cause selective pressure and lead to resistance [34]. Cross-resistance between erythromycin and tylosin could also contribute to the increase in the proportion of macrolide-resistant isolates. Further, Noh et al. [15] and Yoon et al. [45] have identified the *erm*(*A*) and *erm*(*B*) genes in virulent and multi-resistant strains of *E. faecalis* from chickens in Korea, indicating the dissemination of macrolide resistance genes among enterococcal isolates.

The quinupristin/dalfopristin resistance rate in *E. faecium* isolated from chickens was consistent with Kim et al. [16] in Korea and Unal et al. [32] in Canada, but much lower than other studies reported in Canada (89.7%) [30] and the United States (63%) [31]. The quinupristin/dalfopristin resistance rate in *E. faecium* isolated from pigs and cattle was comparable to the findings of Ramos et al. [28] in Portugal. Quinupristin/dalfopristin is not approved for animal use in Korea. The use of virginiamycin, a streptogramin showing cross-resistance with quinupristin/dalfopristin, could be linked to the occurrence of quinupristin/dalfopristin resistance in *E. faecium* [46]. For *E. faecalis*, the situation appears different, because this bacterium is intrinsically resistant to quinupristin/dalfopristin through activity conferred by the expression of the *lsa* gene [39]. Quinupristin/dalfopristin-resistant *Enterococcus* in food animals may play a role in the emergence of human infections through the food chain, indicating the need for continuous and careful monitoring.

We also investigated antibiotic susceptibility to four antibiotics, namely daptomycin, tigecycline, linezolid and vancomycin, representing four different classes and not registered for use in veterinary medicine in Korea [34]. Consistent with previous reports in Korea [16,17,37,38] and other countries [47,48,49,50], the occurrence of linezolid resistance is still rare among enterococcal isolates from food animals. The rarity of linezolid resistance among enterococci might be due to the fact that resistance develops as a spontaneous mutation in the multiple copies *23S rRNA* gene [48]. A small proportion (≤10%) of *E. faecium* and *E. faecalis* isolates exhibited resistance to daptomycin and tigecycline, which are considered critical to humans. Daptomycin resistance in *E. faecium* and *E. faecalis* is typically coordinated by the three-component cell envelope stress response system, LiaFSR [51]. The daptomycin resistance rate found in this study agreed with previous reports in enterococcal isolates from ducks and food animal carcasses in Korea [17,38] and cattle in Australia [10]. However, the tigecycline resistance rate contradicts the above reports. Vancomycin resistance in *E. faecium* and *E. faecalis* isolated from food animals has been reported in many countries [52]. In this study; however, all of the enterococcal isolates were susceptible to vancomycin. Overall, enterococcal isolates resistant to newer and critically important antimicrobials might transfer to humans through the food chain and makes the treatment of MDR infections a daunting clinical challenge.

The majority of *E. faecium* and *E. faecalis* isolates were resistant to at least one antimicrobial agent, and numerous resistance patterns were found in both species. The MDR rates in *E. faecium* and *E. faecalis* isolated from pigs and chickens were higher than those found by Kwon et al. [18] in Korea. In contrast, the MDR rate in *Enterococcus* isolates from chickens, pigs, and cattle from this study contradicts various reports in Europe [29,32] and other Asian countries [13,26]. Consistent with Novais et al. [6], Kim et al. [16] and Kwon et al. [18], MDR patterns usually include tetracycline, erythromycin and/or ciprofloxacin. The occurrence of MDR in *Enterococcus* species might be related to the propensity of the bacteria to be involved in various forms of conjugation. This can lead to the widespread dissemination of resistance determinants through plasmids [53]. Additionally, the hardiness of enterococci species may likely contribute to resistance development by enhancing the survivability of MDR strains in the environment. This has the potential of enhancing transmission from animals to humans [53]. Multidrug-resistant enterococcal isolates pose a serious threat to public health as the same class of antibiotics is being used in the treatment of most bacterial diseases in humans.

In conclusion, we found enterococcal isolates that exhibited resistance to several antimicrobials, including those considered critical for humans. The occurrence of a high percentage of multidrug-resistant *E. faecium* and *E. faecalis* in food animals is alarming, especially given the fact that very few antimicrobial agents can be used to control enterococcal infection. Such resistance is likely to be passed from food animals to humans through the food chain. Therefore, the prudent use of antimicrobials in food animals will be crucial in limiting the public health hazards of *Enterococcus* in Korea.

## Figures and Tables

**Table 1:** Antimicrobial resistance rate in *E. faecium* (*n* = 3360) and *E. faecalis (n =* 4218*)* recovered from cattle, pigs, and chickens between 2010 and 2019 in Korea.

	Resistance Rate % (Number of Isolates)							
	*E. faecium*				*E. faecalis*			
Antimicrobials	Cattle	Pigs	Chickens	Subtotal	Cattle	Pigs	Chickens	Subtotal
	(*n* = 572)	(*n* = 1385)	(*n* = 1403)	(*n* = 3360)	(*n* = 910)	(*n* = 1556)	(*n* = 1752)	(*n* = 4218)
Ampicillin	0.3 (2)	0.1 (1) ^†^	11.4 (160) ^#^	4.8 (163)	0 (0)	0.1 (1)	0.1 (2)	0.1 (3)
Chloramphenicol	2.3 (13) *	6.9 (95) ^†^	18.8 (264) ^#^	11.1 (372)	12.1 (110) *	50.0(778) ^†^	20.9 (367) ^#^	29.8 (1255)
Ciprofloxacin	23.1 (132) *	13.6 (189) ^†^	67.9 (953) ^#^	37.9 (1274)	7.9 (72) *	13.7 (213) ^†^	48.6 (852^) #^	27.0 (1137)
Daptomycin	5.6 (32)	6.9 (95) ^†^	11.1 (156) ^#^	8.4 (283)	1.3 (12)	0.6 (10)	0.6 (10) ^#^	0.8 (32)
Erythromycin	19.9 (114) *	31.5 (436) ^†^	59.5 (835) ^#^	41.2 (1385)	20.4 (186) *	67.1 (1044) ^†^	63.0 (1104) ^#^	55.3 (2334)
Florfenicol	1.6 (9) *	8.4 (116) ^†^	18.7 (262) ^#^	11.5 (387)	4.5 (41) *	45.8 (712) ^†^	14.3 (250) ^#^	23.8 (1003)
Gentamicin	0.2 (1)	0.7 (10) ^†^	2.4 (33) ^#^	1.3 (44)	7.5 (68) *	18.9 (294) ^†^	10.3 (181) ^#^	12.9 (543)
Kanamycin	6.3 (36) *	9.5 (132)	10.1 (142) ^#^	9.2 (310)	13.4 (122) *	44.3 (690) ^†^	21.3 (374) ^#^	28.1 (1186)
Linezolid	0.3 (2)	0 (0) ^†^	5.2 (73) ^#^	2.2 (75)	0.2 (2) *	1.4 (22)	1.1 (20) ^#^	1.0 (44)
Quinupristin/dalfopristin	8.7 (50)	11.9 (165) ^†^	28.0 (393) ^#^	18.1 (608)	ND	ND	ND	ND
Salinomycin	0.2 (1)	0.4 (5) ^†^	11.0(154) ^#^	4.8 (160)	0 (0)	0 (0) ^†^	2.8 (49) ^#^	1.2 (49)
Streptomycin	6.8 (39) *	12.4 (172) ^†^	30.8 (432) ^#^	19.1 (643)	18.8 (171) *	50.9 (792) ^†^	31.8 (558) ^#^	36.1 (1521)
Tetracycline	25.9 (148) *	20.1 (279) ^†^	61.7 (866) ^#^	38.5 (1293)	41.8 (380) *	78.3 (1219)	76.1 (1333) ^#^	69.5 (2932)
Tigecycline	6.8 (39)	5.8 (81)	5.5 (77)	5.9 (197)	7.5 (68) *	11.5 (179)	10.6 (185) ^#^	10.2 (432)
Tylosin	4.9 (28) *	13.8 (191) ^†^	53.0 (743) ^#^	28.6 (962)	20.1 (183) *	66.6 (1036)	63.6 (1115) ^#^	55.3 (2334)
Vancomycin	0 (0)	0 (0)	0 (0)	0 (0)	0 (0)	0 (0)	0 (0)	0 (0)
MDR	8.6 (49) *	15.6 (216) ^†^	60.9 (854) ^#^	33.3 (1119)	19.7 (179) *	63.6 (990) ^†^	59.7 (1046) ^#^	52.5 (2215)

* p < 0.05 compared with the resistance rates in pigs, ^#^ *p* < 0.05 compared with the resistance rates in chickens, and ^†^ *p* < 0.05 compared with the resistance rates in pigs.

**Table 2:** Frequent resistance patterns in *E. faecium* isolates (*n* = 3360) recovered from cattle, pigs, and chickens between 2010 and 2019 in Korea.

Animal Species	No. of Antimicrobials	No. of Isolates (%)	Most Frequent Resistance Pattern
Cattle (*n* = 572)	0	216 (37.8)	
	1	199 (34.8)	ERY (*n* = 63)
	2	107 (18.7)	CIP TET (*n* = 43)
	3	22 (3.8)	CHL STR TET (*n* = 3)
	4	7 (1.2)	ERY STR TET TYL (*n* = 4)
	5	3 (0.5)	CIP ERY KAN TET TYL (*n* = 1), ERY KAN STR TET TYL (*n* = 1), ERY SYN STR TET TYL (*n* = 1)
	6	9 (1.6)	CIP ERY KAN STR TET TYL (*n* = 3)
	7	4 (0.7)	AMP CHL CIP ERY STR TET TYL (*n* = 1), CHL CIP FFC ERY STR TET TYL (*n* = 1), CHL FFC ERY KAN STR TET TYL (*n* = 1), CIP ERY KAN SYN STR TET TYL (*n* = 1)
	8	4 (0.7)	CHL CIP FFC ERY KAN STR TET TYL (*n* = 1), CHL CIP FFC ERY LZD STR TET TYL (*n* = 1), CHL CIP FFC ERY SYN STR TET TYL (*n* = 1), CHL FFC ERY KAN SYN STR TET TYL (*n* = 1)
	9	0 (0)	
	10	1 (0.2)	AMP CHL CIP FFC ERY KAN SYN STR TET TYL (*n* = 1)
Pigs (*n* = 1385)	0	605 (43.7)	
	1	406 (29.3)	ERY (*n* = 179)
	2	137 (9.9)	CIP TET (*n* = 25)
	3	49 (3.5)	ERY TET TYL (*n* = 8)
	4	26 (1.9)	CHL FFC ERY TYL (*n* = 3), CIP ERY TET TYL (*n* = 3), ERY STR TET TYL (*n* = 3)
	5	44 (3.2)	ERY KAN STR TET TYL (*n* = 9)
	6	56 (4.0)	CHL FFC ERY STR TET TYL (*n* = 17)
	7	28 (2.0)	CHL FFC ERY KAN STR TET TYL (*n* = 8)
	8	25 (1.8)	CHL FFC ERY KAN SYN STR TET TYL (*n* = 13)
	9	6 (0.4)	CHL CIP FFC ERY KAN SYN STR TET TYL (*n* = 4)
	10	3 (0.2)	AMP CHL CIP FFC DAP ERY SYN STR TET TYL (*n* = 1), CHL CIP FFC ERY GEN KAN SYN STR TET TYL (*n* = 1), CHL CIP FFC ERY KAN SYN STR TET TGC TYL (*n* = 1)
Chicken (*n* = 1403)	0	134 (9.6)	
	1	205 (14.6)	CIP (*n* = 80)
	2	158 (11.3)	CIP TET (*n* = 73)
	3	132 (9.4)	CIP STR TET (*n* = 24)
	4	146 (10.4)	CIP ERY TET TYL (*n* = 53)
	5	195 (13.9)	CIP ERY STR TET TYL (*n* = 48)
	6	191 (13.6)	CIP ERY SYN STR TET TYL (*n* = 38)
	7	101 (7.2)	AMP CIP ERY SYN STR TET TYL (*n* = 7)
	8	73 (5.2)	CHL CIP FFC ERY SYN STR TET TYL (*n* = 12)
	9	51 (3.6)	AMP CHL CIP FFC ERY SYN STR TET TYL (*n* = 8)
	10	16 (1.1)	AMP CHL CIP FFC ERY LZD SAL STR TET TYL (*n* = 3)
	11	1 (0.1)	CHL CIP FFC ERY KAN LZD SYN SAL STR TET TYL (*n* = 1)

Abbreviations: AMP, Ampicillin; CIP, Ciprofloxacin; CHL, Chloramphenicol; DAP, Daptomycin; ERY, Erythromycin; FFC, Florfenicol; GEN, Gentamicin; KAN, Kanamycin; LNZ, Linezolid; SAL, Salinomycin; STR, Streptomycin; SYN, Quinupristin/Dalfopristin; TET, Tetracycline; TYL, Tylosin; TGC, Tigecycline; VA, Vancomycin.

**Table 3:** Frequent resistance patterns in *E. faecalis* isolates (*n* = 4218) recovered from cattle, pigs, and chickens between 2010 and 2019 in Korea.

Animal Species	No. of Antimicrobials	No. of Isolates (%)	Most Frequent Resistance Pattern
Cattle (*n* = 910)	0	454 (49.9)	
	1	184 (20.2)	TET (*n* = 132)
	2	66 (7.3)	STR TET (*n* = 32)
	3	46 (5.1)	ERY TET TYL (*n* = 19)
	4	28 (3.1)	CHL ERY TET TYL (*n* = 10)
	5	32 (3.5)	ERY KAN STR TET TYL (*n* = 17)
	6	33 (3.6)	ERY GEN KAN STR TET TYL (*n* = 7)
	7	47 (5.2)	CHL ERY GEN KAN STR TET TYL (*n* = 24)
	8	18 (2.0)	CHL CIP ERY GEN KAN STR TET TYL (*n* = 6)
	9	2 (0.2)	CHL CIP FFC ERY KAN LZD STR TET TYL (*n* = 1),
			CHL FFC DAP ERY GEN KAN STR TET TYL (*n* = 1)
Pigs (*n* = 1556)	0	216 (13.9)	
	1	186 (12.0)	TET (*n* = 139)
	2	79 (5.1)	STR TET (*n* = 24)
	3	88 (5.7)	ERY TET TYL (*n* = 45)
	4	104 (6.7)	CHL ERY TET TYL (*n* = 20)
	5	133 (8.5)	CHL FFC ERY TET TYL (*n* = 53)
	6	269 (17.3)	CHL FFC ERY STR TET TYL (*n* = 67)
	7	248 (15.9)	CHL FFC ERY KAN STR TET TYL (*n* = 147)
	8	156 (10.0)	CHL FFC ERY GEN KAN STR TET TYL (*n* = 73)
	9	66 (4.2)	CHL CIP FFC ERY GEN KAN STR TET TYL (*n* = 37)
	10	11 (0.7)	CHL CIP FFC ERY GEN KAN STR TET TGC TYL (*n* = 6)
Chickens (*n* = 1752)	0	144 (8.2)	
	1	264 (15.1)	TET (*n* = 202)
	2	143 (8.2)	CIP TET (*n* = 39)
	3	222 (12.7)	ERY TET TYL (*n* = 83)
	4	369 (21.1)	CIP ERY TET TYL (*n* = 193)
	5	277 (15.8)	CIP ERY STR TET TYL (*n* = 52)
	6	141 (8.0)	CIP ERY GEN KAN TET TYL (*n* = 17)
	7	97 (5.5)	CHL CIP ERY KAN STR TET TYL (*n* = 16)
	8	64 (3.7)	CHL CIP ERY GEN KAN STR TET TYL (*n* = 23)
	9	24 (1.4)	CHL CIP FFC ERY GEN KAN STR TET TYL (*n* = 14)
	10	7 (0.4)	CHL CIP FFC ERY GEN KAN LZD STR TET TYL (*n* = 4)

Abbreviations: AMP, Ampicillin; CIP, Ciprofloxacin; CHL, Chloramphenicol; DAP, Daptomycin; ERY, Erythromycin; FFC, Florfenicol; GEN, Gentamicin; KAN, Kanamycin; LNZ, Linezolid; SAL, Salinomycin; STR, Streptomycin; TET, Tetracycline; TYL, Tylosin; TGC, Tigecycline; VA, Vancomycin.

## Data Availability

The data that support the findings of this study are available from the corresponding author upon reasonable request.

## Supplementary Materials

The following are available online at https://www.mdpi.com/article/10.3390/microorganisms9050925/s1. Table S1. *E. faecium* and *E. faecalis* isolates recovered from cattle, pigs, and chickens between 2010 and 2019 in Korea. Table S2. MIC distribution of the tested antimicrobials against *E. faecium* isolated from cattle, chickens, and pigs between 2010 and 2019 in Korea. Table S3. The MIC_50_ and MIC_90_ of the tested antimicrobials against *E. faecium* isolated from cattle (A), pigs (B), chickens (C) between 2010 and 2019 in Korea. Table S4. MIC distribution of the tested antimicrobials against *E. faecalis* isolated from cattle, chickens, and pigs between 2010 and 2019 in Korea. Table S5 A. The MIC_50_ and MIC_90_ of the tested antimicrobials against *E. faecalis* isolated from cattle (A), pigs (B), chickens (C) between 2010 and 2019 in Korea. Table S6. Antimicrobial resistance rate in *E. faecium* isolated from cattle between 2010 and 2019 in Korea. Table S7. Antimicrobial resistance rate in *E. faecium* isolated from pigs between 2010 and 2019 in Korea. Table S8. Antimicrobial resistance rate in *E. faecium* isolated from chickens between 2010 and 2019 in Korea. Table S9. Antimicrobial resistance rate in *E. faecalis* isolated from cattle between 2010 and 2019 in Korea. Table S10. Antimicrobial resistance rate in *E. faecalis* isolated from pigs between 2010 and 2019 in Korea. Table S11. Antimicrobial resistance rate in *E. faecalis* isolated from chickens between 2010 and 2019 in Korea.
