# Supplementary material for: Nationwide Surveillance on Antimicrobial Resistance Profiles of Enterococcus faecium and Enterococcus faecalis Isolated from Healthy Food Animals in South Korea, 2010 to 2019
Source: Microorganisms. 2021 Apr 26;9(5):925. doi: 10.3390/microorganisms9050925 (PMC8144984; doi:10.3390/microorganisms9050925)
Supplement: Supplementary file 1 [file microorganisms-09-00925-s001.zip › microorganisms-1194159-supplementary.pdf]

Table S1. *Enterococcus faecium* and *Enterococcus faecalis* isolates recovered from cattle, pigs, and chickens between 2010 and 2019 in Korea.

Year	*E. faecium*									*E. faecalis*								
	**Cattle**			**Pigs**			**Chickens**			**Cattle**			**Pigs**			**Chickens**		
	**No. of slaughter houses**	**No. of farms**	**No. of isolates**	**No. of slaughter houses**	**No. of farms**	**No. of isolates**	**No. of slaughter houses**	**No. of farms**	**No. of isolates**	**No. of slaughter houses**	**No. of farms**	**No. of isolates**	**No. of slaughter houses**	**No. of farms**	**No. of isolates**	**No. of slaughter houses**	**No. of farms**	**No. of isolates**
2010	10	16	25	17	49	60	19	77	77	18	57	61	19	74	88	20	157	162
2011	17	70	72	27	114	163	12	78	87	24	137	152	30	142	216	12	130	170
2012	18	48	53	27	123	148	12	147	168	21	75	80	25	123	145	12	175	213
2013	17	46	46	28	125	127	14	132	134	17	84	85	27	158	166	15	192	196
2014	14	53	73	26	116	144	13	184	240	17	79	98	25	129	164	12	195	229
2015	14	50	51	23	132	142	11	150	166	20	101	103	21	165	173	12	167	175
2016	17	51	57	23	136	152	11	139	162	19	76	95	24	167	200	13	159	181
2017	17	59	60	23	127	139	13	90	90	13	73	76	25	147	155	13	134	138
2018	22	76	78	24	154	197	19	145	168	19	79	80	24	138	143	21	153	154
2019	18	56	57	24	111	113	16	107	111	22	80	80	22	104	106	21	130	134
Total	46*	512	572	59	1045	1385	40	1086	1403	53	830	910	59	1154	1556	45	1374	1752

*No. of different slaughterhouses.

Table S2. MIC distribution of the tested antimicrobials against *E. faecium* (n=3360) isolated from cattle, chickens, and pigs between 2010 and 2019 in Korea.

Antimicrobials	Source	MIC (μg/mL) distribution (%)															MIC_{50}	MIC_{90}
		0.12	0.25	0.5	1	2	4	8	16	32	64	128	256	512	1024	2048		
Ampicillin	Cattle				74.7	21.7	2.8	0.5	0.3								1	2
	Pigs				67.0	23.4	9.0	0.5	0.1								1	2
	Chickens				36.6	15.6	29.9	6.5	11.4								2	16
Chloramphenicol	Cattle					4.4	34.3	57.9	1.2	2.3							8	8
	Pigs					1.0	33.1	56.0	3.0	6.9							8	8
	Chickens					2.1	18.7	55.5	4.9	18.8							8	32
Ciprofloxacin	Cattle		2.6	14.5	33.2	26.6	17.8	4.9	0.3								1	4
	Pigs		1.7	16.2	47.7	20.8	9.5	3.1	1.0								1	4
	Chickens		0.2	3.6	11.6	16.7	26.7	16.7	24.5								4	16
Daptomycin	Cattle			4.7	2.8	21.7	65.2	4.7	0.3	0.5							4	4
	Pigs			4.5	5.9	23.9	58.8	6.7	0	0.1							4	4
	Chickens			3.8	4.8	26.4	53.8	10.4	0.4	0.3							4	8
Erythromycin	Cattle				32.5	15	32.5	13.1	2.1	0	4.7						4	8
	Pigs				20.0	10.6	37.9	16.0	2.9	0.6	12.0						4	64
	Chickens				21.7	9.6	9.3	6.4	1.9	1.4	49.8						32	64
Florfenicol	Cattle					43.9	54.5	0	0.2	1.4							4	4
	Pigs					39.1	52.3	0.2	0.9	7.5							4	4
	Chickens					28.7	51.2	1.4	0.6	18							4	32
Gentamicin	Cattle											99.8	0	0	0.2	0	128	128
	Pigs											98.6	0.7	0	0.1	0.6	128	128
	Chickens											97.0	0.6	0.1	0.4	2.0	128	128
Kanamycin	Cattle											59.3	27.8	6.6	1.0	5.2	128	512
	Pigs											53.7	30.4	6.4	0.7	8.8	128	512
	Chickens											56.0	29.0	4.9	0.9	9.2	128	1024

Linezolid	Cattle			0.9	8.0	82.3	8.4	0.3	0								2	2
	Pigs			0.2	6.9	80.8	12.1	0	0								2	4
	Chickens			0.6	11.7	66.9	15.5	4.1	1.1								2	4
Quinupristin/ dalfopristin	Cattle				30.4	60.8	8.2	0.3	0	0.2							2	2
	Pigs				19.3	68.8	10.7	1.0	0.1	0.1							2	4
	Chickens				23.3	48.7	19.2	4.1	2.1	2.6							2	4
Salinomycin	Cattle					97.0	2.8	0.2	0	0							2	2
	Pigs					96.2	3.4	0.4	0	0							2	2
	Chickens					68.0	21.0	10.8	0.2	0							2	8
Streptomycin	Cattle											92.7	0.3	0.2	1.2	5.6	128	256
	Pigs											86.5	0.5	0.6	1.2	11.3	128	2048
	Chickens											62.9	3.0	3.3	3.3	27.5	128	2048
Tetracycline	Cattle					72.9	1.0	0.2	0.7	4.0	5.1	16.1					2	128
	Pigs					78.3	1.2	0.4	0.8	0.8	3.0	15.5					2	128
	Chickens					37.3	0.4	0.5	0.6	1.9	6.6	52.6					128	128
Tigecycline	Cattle	67.8	25.3	6.5	0.3	0											0.12	0.25
	Pigs	71.3	22.8	5.5	0.4	0											0.12	0.25
	Chickens	64.9	29.7	5.2	0.2	0.1											0.12	0.25
Tylosin	Cattle				4.4	26.7	35.3	27.4	1.2	0.2	4.7						4	8
	Pigs				2.9	17.5	39.9	25.4	0.4	0	13.8						4	64
	Chickens				4.3	15.0	18.2	8.3	1.2	0.3	52.7						64	64
Vancomycin	Cattle					96.7	3.1	0.2	0	0							2	2
	Pigs					97.9	2.1	0	0	0							2	2
	Chickens					97.6	2.4	0	0.1	0							2	2

The breakpoints of tested antimicrobial agents are indicated by vertical lines.

MIC_{50} and MIC_{90} are the concentrations (μg/mL) at which 50% and 90% of the isolates were inhibited, respectively.

Table S3A. The MIC_{50} and MIC_{90} of the tested antimicrobials against *E. faecium* (n=572) isolated from cattle between 2010 and 2019 in Korea.

**Antimicrobials**		**2010 (n=25)**	**2011 (n=72)**	**2012 (n=53)**	**2013 (n=46)**	**2014 (n=73)**	**2015 (n=51)**	**2016 (n=57)**	**2017 (n=60)**	**2018 (n=78)**	**2019 (n=57)**
Ampicillin	MIC_{50}	1	1	1	1	1	1	1	1	1	1
	MIC_{90}	4	2	1	2	2	2	2	2	2	2
	Resistance (%)	0	0	0	2.2	0	0	0	0	0	1.8
Chloramphenicol	MIC_{50}	4	4	8	8	8	8	8	4	8	8
	MIC_{90}	8	8	8	8	8	8	8	8	8	8
	Resistance (%)	8.0	1.4	0	2.2	2.7	5.9	1.8	0	1.3	3.5
Ciprofloxacin	MIC_{50}	1	1	2	2	1	1	2	1	1	2
	MIC_{90}	4	2	4	4	4	4	4	4	4	8
	Resistance (%)	20.0	9.7	15.1	17.4	16.4	25.5	43.9	28.3	21.8	35.1
Daptomycin	MIC_{50}	2	2	4	4	4	4	4	4	4	4
	MIC_{90}	4	4	4	4	4	4	8	4	4	8
	Resistance (%)	0	2.8	1.9	6.5	2.7	3.9	19.3	1.7	5.1	10.5
Erythromycin	MIC_{50}	4	2	2	4	2	2	2	4	4	4
	MIC_{90}	64	8	8	4	4	4	4	8	8	8
	Resistance (%)	40.0	30.6	13.2	6.5	6.8	9.8	8.8	30	34.6	21.1
Florfenicol	MIC_{50}	2	2	4	4	4	4	4	2	4	4
	MIC_{90}	4	2	4	4	4	4	4	4	4	4
	Resistance (%)	8.0	2.8	0	0	2.7	0	0	1.7	1.3	1.8
Gentamicin	MIC_{50}	128	128	128	128	128	128	128	128	128	128
	MIC_{90}	128	128	128	128	128	128	128	128	128	128
	Resistance (%)	4.0	0	0	0	0	0	0	0	0	0
Kanamycin	MIC_{50}	128	256	128	128	256	128	128	128	128	128
	MIC_{90}	2048	2048	512	512	256	512	256	256	256	256
	Resistance (%)	12.0	26.4	1.9	8.7	2.7	3.9	0	3.3	1.3	3.5
Linezolid	MIC_{50}	2	2	2	2	2	2	2	2	2	2
	MIC_{90}	2	2	2	4	2	4	4	2	4	2
	Resistance (%)	0	0	0	0	1.4	0	1.8	0	0	0
Quinupristin/dalfopristin	MIC_{50}	2	2	2	2	2	2	2	2	2	2
	MIC_{90}	4	2	4	4	2	4	2	2	2	4
	Resistance (%)	16.0	1.4	17.0	19.6	8.2	15.7	0	1.7	6.4	12.3
Salinomycin	MIC_{50}	2	2	2	2	2	2	2	2	2	2
	MIC_{90}	4	2	2	2	4	2	2	2	2	2
	Resistance (%)	0	0	1.9	0	0	0	0	0	0	0

Streptomycin	MIC_{50}	128	128	128	1	128	128	128	128	128	128
	MIC_{90}	2048	1024	128	2	128	256	128	128	128	128
	Resistance (%)	32.0	11.1	5.7	6.5	8.2	9.8	1.8	1.7	1.3	5.3
Tetracycline	MIC_{50}	64	4	2	2	2	2	2	2	2	2
	MIC_{90}	128	128	128	64	128	128	128	64	2	64
	Resistance (%)	64.0	47.2	30.2	15.2	26.0	21.6	35.1	16.7	9.0	14.0
Tigecycline	MIC_{50}	0.125	0.125	0.125	0.125	0.125	0.25	0.25	0.12	0.25	0.12
	MIC_{90}	0.25	0.125	0.125	0.125	0.25	0.25	0.5	0.5	0.5	0.5
	Resistance (%)	4.0	1.4	1.9	0	1.4	7.8	10.5	11.7	15.4	10.5
Tylosin	MIC_{50}	4	4	4	8	4	4	4	4	4	4
	MIC_{90}	64	8	8	8	8	8	8	8	8	8
	Resistance (%)	32.0	8.3	3.8	4.3	4.1	2.0	1.8	3.3	1.3	3.5
Vancomycin	MIC_{50}	2	2	2	2	2	2	2	2	2	2
	MIC_{90}	2	2	2	2	4	2	2	2	2	2
	Resistance (%)	0	0	0	0	0	0	0	0	0	0

MIC_{50} and MIC_{90} are the concentrations (μg/mL) at which 50% and 90% of the isolates were inhibited, respectively.

Table S3B. The MIC_{50} and MIC_{90} of the tested antimicrobials against *E. faecium* (n=1385) isolated from pigs between 2010 and 2019 in Korea.

**Antimicrobials**		**2010 (n=60)**	**2011 (n=163)**	**2012 (n=148)**	**2013 (n=127)**	**2014 (n=144)**	**2015 (n=142)**	**2016 (n=152)**	**2017 (n=139)**	**2018 (n=197)**	**2019 (n=113)**
Ampicillin	MIC_{50}	1	1	1	1	1	1	1	2	1	1
	MIC_{90}	4	2	2	2	4	4	4	2	2	4
	Resistance (%)	0	0	0	0.8	0	0	0	0	0	0
Chloramphenicol	MIC_{50}	4	4	8	8	8	8	8	4	8	8
	MIC_{90}	8	8	8	8	32	32	16	16	8	8
	Resistance (%)	5.0	2.5	5.4	3.9	11.1	12.0	6.6	7.2	7.1	7.1
Ciprofloxacin	MIC_{50}	1	1	1	1	1	1	2	1	1	1
	MIC_{90}	4	2	4	4	4	2	4	4	4	4
	Resistance (%)	23.3	6.7	12.2	11.0	11.8	9.2	29.6	12.9	12.2	13.3
Daptomycin	MIC_{50}	2	2	4	4	4	4	4	4	4	4
	MIC_{90}	4	4	4	4	4	4	8	4	4	8
	Resistance (%)	0	0	8.1	3.1	2.8	7.7	25.7	1.4	5.6	10.6
Erythromycin	MIC_{50}	4	4	4	4	4	4	4	4	4	4
	MIC_{90}	64	8	64	64	64	64	64	64	64	64
	Resistance (%)	33.3	25.8	16.9	12.6	15.3	34.5	43.4	38.8	48.2	41.6
Florfenicol	MIC_{50}	2	2	4	4	4	4	4	2	4	4
	MIC_{90}	4	2	4	4	4	32	8	16	4	4
	Resistance (%)	10.0	4.9	8.1	3.9	8.3	13.4	9.2	10.8	8.6	7.1
Gentamicin	MIC_{50}	128	128	128	128	128	128	128	128	128	128
	MIC_{90}	128	128	128	128	128	128	128	128	128	128
	Resistance (%)	0	1.2	0.7	1.6	1.4	0	1.3	0	0	0.9
Kanamycin	MIC_{50}	128	128	128	128	256	128	256	128	128	128
	MIC_{90}	2048	1024	512	512	512	512	1024	256	512	2048
	Resistance (%)	21.7	10.4	7.4	9.4	8.3	7.7	11.8	6.5	8.6	10.6
Linezolid	MIC_{50}	2	2	2	2	2	2	2	2	2	2
	MIC_{90}	2	2	4	4	2	4	4	2	2	2
	Resistance (%)	0	0	0	0	0	0	0	0	0	0
Quinupristin/dalfopristin	MIC_{50}	2	2	2	2	2	2	2	2	2	2
	MIC_{90}	2	2	4	4	4	4	2	2	4	4
	Resistance (%)	8.3	3.1	25.0	11.8	11.1	17.6	9.2	5.0	10.7	17.7
Salinomycin	MIC_{50}	2	2	2	2	2	2	2	2	2	2
	MIC_{90}	2	2	2	2	4	2	4	2	2	2

	Resistance (%)	0	0	0.7	0.8	0.7	0	0.7	0	0.5	0
Streptomycin	MIC_{50}	128	128	128	1	128	128	128	128	128	128
	MIC_{90}	2048	256	2048	2	1024	2048	2048	2048	1024	2048
	Resistance (%)	28.3	7.4	12.8	11.8	11.1	14.8	12.5	12.2	10.7	13.3
Tetracycline	MIC_{50}	2	2	2	2	2	2	2	2	2	2
	MIC_{90}	128	128	128	128	128	128	128	128	64	128
	Resistance (%)	36.7	19.0	23.0	15.0	19.4	21.1	27.0	17.3	16.2	15.9
Tigecycline	MIC_{50}	0.125	0.125	0.125	0.125	0.125	0.125	0.125	0.12	0.25	0.12
	MIC_{90}	0.25	0.125	0.25	0.125	0.25	0.25	0.25	0.25	0.5	0.25
	Resistance (%)	10.0	0.6	2.0	3.1	0.7	2.8	7.2	5.8	17.3	8.0
Tylosin	MIC_{50}	4	4	4	4	4	4	4	4	4	4
	MIC_{90}	64	8	64	64	64	64	64	64	64	64
	Resistance (%)	23.3	7.4	12.8	11.0	12.5	16.2	14.5	13.7	17.3	14.2
Vancomycin	MIC_{50}	2	2	2	2	2	2	2	2	2	2
	MIC_{90}	2	2	2	2	2	2	2	2	2	2
	Resistance (%)	0	0	0	0	0	0	0	0	0	0

MIC_{50} and MIC_{90} are the concentrations (µg/mL) at which 50% and 90% of the isolates were inhibited, respectively.

Table S3C. The MIC_{50} and MIC_{90} of the tested antimicrobials against *E. faecium* isolated from chickens (n=1403) between 2010 and 2019 in Korea.

**Antimicrobials**		**2010 (n=77)**	**2011 (n=87)**	**2012 (n=168)**	**2013 (n=134)**	**2014 (n=240)**	**2015 (n=166)**	**2016 (n=162)**	**2017 (n=90)**	**2018 (n=168)**	**2019 (n=111)**
Ampicillin	MIC_{50}	4	2	2	2	4	4	2	2	2	4
	MIC_{90}	16	16	8	16	8	16	8	16	16	16
	Resistance (%)	13.0	12.6	9.5	10.4	8.3	12.7	4.3	12.2	13.7	24.3
Chloramphenicol	MIC_{50}	8	8	8	8	8	8	8	4	8	8
	MIC_{90}	32	32	32	32	32	32	32	32	32	32
	Resistance (%)	22.1	19.5	16.1	15.7	19.2	14.5	14.8	12.2	26.2	29.7
Ciprofloxacin	MIC_{50}	4	4	4	8	4	4	4	4	4	4
	MIC_{90}	16	16	16	16	16	16	16	16	16	16
	Resistance (%)	74.0	75.9	66.7	70.9	68.3	78.9	65.4	62.2	56.5	64.0
Daptomycin	MIC_{50}	2	2	4	4	4	4	4	2	4	4
	MIC_{90}	4	4	8	4	4	8	8	4	8	8
	Resistance (%)	0	1.1	13.1	8.2	9.6	11.4	19.8	6.7	11.9	19.8
Erythromycin	MIC_{50}	64	64	64	64	64	32	8	4	4	16
	MIC_{90}	64	64	64	64	64	64	64	64	64	64
	Resistance (%)	84.4	74.7	55.4	67.2	59.6	57.8	53.7	50.0	45.2	67.6
Florfenicol	MIC_{50}	2	2	4	4	4	4	4	2	4	4
	MIC_{90}	32	32	32	32	32	32	32	32	32	32
	Resistance (%)	24.7	21.8	14.9	14.2	18.8	15.1	13.0	12.2	26.2	30.6
Gentamicin	MIC_{50}	128	128	128	128	128	128	128	128	128	128
	MIC_{90}	1024	128	128	128	128	128	128	128	128	128
	Resistance (%)	10.4	1.1	3.6	0	1.7	2.4	1.9	2.2	0	4.5
Kanamycin	MIC_{50}	128	128	256	128	256	128	256	128	128	128
	MIC_{90}	2048	256	2048	512	2048	1024	512	256	256	512
	Resistance (%)	20.8	3.4	12.5	9.7	11.7	11.4	6.2	8.9	7.7	9.9
Linezolid	MIC_{50}	1	2	2	2	2	2	2	2	2	2
	MIC_{90}	4	4	4	4	4	4	4	2	4	8
	Resistance (%)	3.9	0	1.2	1.5	7.1	6.6	6.8	3.3	6.5	11.7
Quinupristin/dalfopristin	MIC_{50}	2	2	2	2	2	2	2	2	2	2
	MIC_{90}	16	32	8	8	8	8	4	2	4	4
	Resistance (%)	39.0	26.4	44.0	34.3	29.2	34.3	24.1	6.7	14.9	20.7
Salinomycin	MIC_{50}	2	2	2	2	2	2	2	2	2	2

	MIC_{90}	4	4	8	4	8	8	8	4	4	4
	Resistance (%)	2.6	6.9	11.3	3.7	24.6	17.5	13.0	2.2	3.0	5.4
Streptomycin	MIC_{50}	1024	512	128	2	128	128	128	128	128	128
	MIC_{90}	2048	2048	2048	16	2048	2048	2048	2048	2048	2048
	Resistance (%)	50.6	46.0	41.1	44.0	30.4	38.0	16.7	23.3	13.1	17.1
Tetracycline	MIC_{50}	128	128	128	128	128	128	4	32	2	2
	MIC_{90}	128	128	128	128	128	128	128	128	128	128
	Resistance (%)	87.0	82.8	70.8	77.6	62.9	63.9	48.8	52.2	42.9	44.1
Tigecycline	MIC_{50}	0.125	0.125	0.125	0.125	0.125	0.25	0.125	0.12	0.25	0.12
	MIC_{90}	0.25	0.25	0.25	0.25	0.25	0.25	0.25	0.25	0.25	0.25
	Resistance (%)	3.9	1.1	1.8	3.0	4.6	9.6	9.3	10.0	5.4	5.4
Tylosin	MIC_{50}	64	64	64	64	64	64	8	4	4	8
	MIC_{90}	64	64	64	64	64	64	64	64	64	64
	Resistance (%)	84.4	74.7	51.8	64.9	58.8	51.2	38.3	41.1	35.7	48.6
Vancomycin	MIC_{50}	2	2	2	2	2	2	2	2	2	2
	MIC_{90}	2	2	2	2	2	2	2	2	2	2
	Resistance (%)	0	0	0	0	0	0	0	0	0	0

MIC_{50} and MIC_{90} are the concentrations (μg/mL) at which 50% and 90% of the isolates were inhibited, respectively.

Table S4. MIC distribution of the tested antimicrobials against *E. faecalis* isolated from cattle, chickens, and pigs between 2010 and 2019 in Korea (n=4218).

Antimicrobials	Source	MIC (μg/mL) distribution (%)															MIC_{50}	MIC_{90}
		0.12	0.25	0.5	1	2	4	8	16	32	64	128	256	512	1024	2048		
Ampicillin	Cattle				89.8	10.2	0	0	0								1	2
	Pigs				89.9	10.0	0.1	0	0.1								1	2
	Chickens				93.4	6.4	0	0.1	0.1								1	1
Chloramphenicol	Cattle					1.1	24.3	60.3	2.2	12.1							8	32
	Pigs					0.4	9.8	31.4	8.4	50.0							16	32
	Chickens					0.5	14.1	60.8	3.6	20.9							8	32
Ciprofloxacin	Cattle		0.3	5.1	45.7	41.0	2.5	0	5.4								1	2
	Pigs		0.3	4.7	54.6	26.7	4.6	1.1	8.0								1	4
	Chickens		0.1	2.3	33.2	15.8	2.3	0.3	46.0								2	16
Daptomycin	Cattle			3.7	33.6	47.9	13.4	1.0	0	0.3							2	4
	Pigs			2.2	36.8	52.1	8.4	0.3	0.1	0.3							2	2
	Chickens			5.0	38.0	51.1	5.3	0.2	0	0.3							2	2
Erythromycin	Cattle				51.2	25.8	2.5	0.8	0.8	0.8	18.1						1	64
	Pigs				22.8	8.0	2.2	5.1	4.6	1.7	55.7						64	64
	Chickens				26.3	8.8	1.9	4.5	6.0	3.9	48.6						32	64
Florfenicol	Cattle					46.6	47.7	1.2	2.1	2.4							4	4
	Pigs					21.7	29.7	2.9	17.7	28.0							4	32
	Chickens					34.2	50.1	1.5	2.3	12.0							4	32
Gentamicin	Cattle											91.1	1.0	0.4	2.0	5.5	128	128
	Pigs											75.4	3.3	2.4	5.6	13.3	128	2048
	Chickens											88.7	0.6	0.3	1.1	9.2	128	1024
Kanamycin	Cattle											71.3	14.9	0.3	0.1	13.3	128	2048
	Pigs											44.0	11.2	0.5	0.3	44.0	256	2048
	Chickens											64.7	13.6	0.3	0.6	20.8	128	2048
Linezolid	Cattle			0.4	27.6	70.2	1.5	0.2	0								2	2
	Pigs			0.4	22.2	63.8	12.1	1.2	0.2								2	4
	Chickens			1.3	33.3	57.9	6.0	0.7	0.4								2	2

Salinomycin	Cattle					98.8	1.2	0	0	0							2	2
	Pigs					99.3	0.7	0	0	0							2	2
	Chickens					83.0	14.2	2.8	0	0							2	4
Streptomycin	Cattle											79.7	1.0	0.5	1.4	17.4	128	2048
	Pigs											47.2	1.2	0.7	1.8	49.1	1024	2048
	Chickens											65.2	1.4	1.5	2.1	29.7	128	2048
Tetracycline	Cattle					57.5	0.8	0	0.7	3.2	19.3	18.6					2	128
	Pigs					21.2	0.3	0.2	0.6	2.1	24.7	50.9					128	128
	Chickens					23.1	0.6	0.2	1.6	3.8	11.6	59.1					128	128
Tigecycline	Cattle	64.5	28.0	6.7	0.8	0											0.12	0.25
	Pigs	59.7	28.8	10.0	1.5	0											0.12	0.5
	Chickens	58.6	30.9	10.0	0.5	0.1											0.125	0.5
Tylosin	Cattle				20.7	54.7	4.3	0.2	0	0.1	20.0						2	64
	Pigs				8.3	22.1	2.1	0.6	0.4	1.0	65.6						64	64
	Chickens				7.6	26.4	1.9	0.2	0.2	0.3	63.4						64	64
Vancomycin	Cattle					95.7	4.3	0	0	0							2	2
	Pigs					92.3	7.6	0.1	0	0							2	2
	Chickens					91.0	9.0	0	0	0							2	2

The breakpoints of tested antimicrobial agents are indicated by vertical lines.

MIC_{50} and MIC_{90} are the concentrations at which 50% and 90% of the isolates were inhibited, respectively.

Table S5A. The MIC_{50} and MIC_{90} of the tested antimicrobials against *E. faecalis* (n= 910) isolated from cattle between 2010 and 2019 in Korea.

**Antimicrobials**		**2010 (n=61)**	**2011 (n=152)**	**2012 (n=80)**	**2013 (n=85)**	**2014 (n=99)**	**2015 (n=102)**	**2016 (n=95)**	**2017 (n=76)**	**2018 (n=80)**	**2019 (n=80)**
Ampicillin	MIC_{50}	1	1	1	1	1	1	1	1	1	1
	MIC_{90}	1	1	1	2	2	2	1	1	1	1
	Resistance (%)	0	0	0	0	0	0	0	0	0	0
Chloramphenicol	MIC_{50}	4	4	8	8	8	8	8	8	8	8
	MIC_{90}	32	32	32	16	32	32	32	16	8	32
	Resistance (%)	16.4	15.1	16.3	3.5	11.1	19.6	10.5	7.9	6.3	11.3
Ciprofloxacin	MIC_{50}	1	1	2	1	2	2	2	1	1	2
	MIC_{90}	2	2	2	2	2	4	4	2	2	2
	Resistance (%)	0	9.9	8.8	9.4	8.1	12.7	13.7	3.9	3.8	2.5
Daptomycin	MIC_{50}	1	1	2	2	2	2	2	2	2	2
	MIC_{90}	2	2	4	2	4	4	4	4	4	4
	Resistance (%)	0	0.7	1.3	0	2.0	2.0	3.2	1.3	1.3	1.3
Erythromycin	MIC_{50}	2	1	1	2	1	1	2	2	2	2
	MIC_{90}	64	64	64	64	64	64	64	64	64	64
	Resistance (%)	24.6	32.2	23.8	17.6	13.1	19.6	23.2	11.8	16.3	13.8
Florfenicol	MIC_{50}	2	2	4	4	4	4	4	2	2	2
	MIC_{90}	2	2	4	4	4	4	8	4	4	4
	Resistance (%)	0	0.7	6.3	3.5	3.0	8.8	8.4	5.3	3.8	6.3
Gentamicin	MIC_{50}	128	128	128	128	128	128	128	128	128	128
	MIC_{90}	2048	256	256	128	128	128	128	128	128	128
	Resistance (%)	13.1	8.6	8.8	7.1	9.1	5.9	7.4	2.6	5.0	7.5
Kanamycin	MIC_{50}	128	128	128	128	256	128	256	128	128	128
	MIC_{90}	2048	2048	2048	2048	2048	2048	2048	256	2048	2048
	Resistance (%)	14.8	15.8	16.3	15.3	11.1	10.8	18.9	5.3	12.5	11.3
Linezolid	MIC_{50}	1	1	2	2	2	2	2	2	2	2
	MIC_{90}	1	2	2	2	2	2	2	2	2	2
	Resistance (%)	0	0	0	0	0	2	0	0	0	0
Salinomycin	MIC_{50}	2	2	2	2	2	2	2	2	2	2
	MIC_{90}	2	2	2	2	2	2	2	2	2	2
	Resistance (%)	0	0	0	0	0	0	0	0	0	0

Streptomycin	MIC_{50}	128	128	128	128	128	128	128	128	128	128
	MIC_{90}	2048	2048	2048	2048	2048	2048	2048	2048	128	128
	Resistance (%)	23.0	19.1	27.5	22.4	24.2	18.6	21.1	11.8	10.0	8.8
Tetracycline	MIC_{50}	32	32	64	2	2	2	2	2	2	2
	MIC_{90}	128	128	128	128	128	128	128	128	64	128
	Resistance (%)	54.1	52.0	58.8	43.5	46.5	42.2	35.8	27.6	22.5	27.5
Tigecycline	MIC_{50}	0.125	0.125	0.125	0.125	0.125	0.125	0.125	0.25	0.25	0.25
	MIC_{90}	0.25	0.125	0.25	0.125	0.25	0.25	0.5	0.5	0.5	0.5
	Resistance (%)	0	0	3.8	1.2	0	7.8	20.0	22.4	12.5	12.5
Tylosin	MIC_{50}	2	2	2	2	2	2	2	2	2	2
	MIC_{90}	64	64	64	64	64	64	64	64	64	64
	Resistance (%)	24.6	31.6	22.5	17.6	13.1	19.6	22.1	11.8	16.3	13.8
Vancomycin	MIC_{50}	2	2	2	2	2	2	2	2	2	2
	MIC_{90}	2	2	2	2	2	2	2	2	2	2
	Resistance (%)	0	0	0	0	0	0	0	0	0	0

MIC_{50} and MIC_{90} are the concentrations (μg/mL) at which 50% and 90% of the isolates were inhibited.

Table S5B. The MIC_{50} and MIC_{90} of the tested antimicrobials against *E. faecalis* (n=1556) *isolated* from pigs between 2010 and 2019 in Korea.

**Antimicrobials**		**2010 (n=88)**	**2011 (n=216)**	**2012 (n=145)**	**2013 (n=166)**	**2014 (n=164)**	**2015 (n=173)**	**2016 (n=200)**	**2017 (n=155)**	**2018 (n=143)**	**2019 (n=106)**
Ampicillin	MIC_{50}	1	1	1	1	1	1	1	1	1	1
	MIC_{90}	1	1	1	2	2	2	2	1	1	1
	Resistance (%)	0	0.5	0	0	0	0	0	0	0	0
Chloramphenicol	MIC_{50}	8	16	16	16	16	32	16	32	32	32
	MIC_{90}	32	32	32	32	32	32	32	32	32	32
	Resistance (%)	45.5	48.6	44.1	48.8	46.3	55.5	41.0	61.3	55.2	56.6
Ciprofloxacin	MIC_{50}	1	1	1	1	1	1	2	1	1	1
	MIC_{90}	2	16	2	4	4	16	4	8	16	2
	Resistance (%)	9.1	13.4	9.7	10.8	12.8	16.8	17.5	15.5	18.9	7.5
Daptomycin	MIC_{50}	1	1	2	2	2	2	2	2	2	2
	MIC_{90}	2	2	2	2	4	4	4	2	4	2
	Resistance (%)	0	0.5	0	1.2	1.2	1.7	0.5	0	0	0.9
Erythromycin	MIC_{50}	64	64	64	64	8	64	16	64	64	64
	MIC_{90}	64	64	64	64	64	64	64	64	64	64
	Resistance (%)	64.8	67.1	59.3	65.1	60.4	70.5	63.5	75.5	74.8	71.7
Florfenicol	MIC_{50}	2	2	4	4	16	16	4	16	16	16
	MIC_{90}	32	32	32	32	32	32	32	32	32	32
	Resistance (%)	23.9	31.5	40.7	42.8	53.7	52.6	43.5	55.5	57.3	55.7
Gentamicin	MIC_{50}	128	128	128	128	128	128	128	128	128	128
	MIC_{90}	2048	2048	2048	2048	2048	2048	1024	2048	2048	2048
	Resistance (%)	22.7	18.1	20.0	17.5	14.0	23.1	12.5	16.8	24.5	26.4
Kanamycin	MIC_{50}	128	256	128	128	256	128	256	256	2048	128
	MIC_{90}	2048	2048	2048	2048	2048	2048	2048	2048	2048	2048
	Resistance (%)	47.7	48.6	44.8	41.0	39.0	47.4	34.5	44.5	52.4	48.1
Linezolid	MIC_{50}	1	1	2	2	2	2	2	2	2	2
	MIC_{90}	1	2	4	4	2	4	4	4	4	4
	Resistance (%)	0	0.5	0.7	0.6	0	2.3	1.0	1.9	2.8	5.7
Salinomycin	MIC_{50}	2	2	2	2	2	2	2	2	2	2
	MIC_{90}	2	2	2	2	2	2	2	2	2	2

	Resistance (%)	0	0	0	0	0	0	0	0	0	0
Streptomycin	MIC_{50}	1024	128	2048	128	256	2048	128	2048	2048	2048
	MIC_{90}	2048	2048	2048	2048	2048	2048	2048	2048	2048	2048
	Resistance (%)	51.1	45.4	51.0	45.8	48.8	60.1	46.5	52.3	59.4	52.8
Tetracycline	MIC_{50}	64	64	64	64	128	128	128	128	64	64
	MIC_{90}	128	128	128	128	128	128	128	128	128	128
	Resistance (%)	75.0	80.6	80.7	77.7	76.2	83.2	73.0	81.9	79.7	72.6
Tigecycline	MIC_{50}	0.125	0.125	0.125	0.125	0.25	0.125	0.25	0.25	0.25	0.25
	MIC_{90}	0.25	0.125	0.25	0.25	0.25	0.25	0.5	0.5	0.5	0.5
	Resistance (%)	0	0.9	4.1	3.0	1.8	9.8	33.5	27.1	16.1	13.2
Tylosin	MIC_{50}	64	64	64	64	2	64	64	64	64	64
	MIC_{90}	64	64	64	64	64	64	64	64	64	64
	Resistance (%)	64.8	67.1	59.3	65.1	59.8	70.5	62.0	73.5	74.1	71.7
Vancomycin	MIC_{50}	2	2	2	2	2	2	2	2	2	2
	MIC_{90}	2	2	2	2	4	4	2	2	2	2
	Resistance (%)	0	0	0	0	0	0	0	0	0	0

MIC_{50} and MIC_{90} are the concentrations (μg/mL) at which 50% and 90% of the isolates were inhibited, respectively.

Table S5C. The MIC_{50} and MIC_{90} of the tested antimicrobials against *E. faecalis* (n=1752) isolated from chickens between 2010 and 2019 in Korea.

**Antimicrobials**		**2010 (n=162)**	**2011 (n=170)**	**2012 (n=213)**	**2013 (n=196)**	**2014 (n=229)**	**2015 (n=175)**	**2016 (n=181)**	**2017 (n=138)**	**2018 (n=154)**	**2019 (n=134)**
Ampicillin	MIC_{50}	1	1	1	1	1	1	1	1	1	1
	MIC_{90}	1	1	1	1	1	2	1	1	1	1
	Resistance (%)	0	0	0	0	0	0.6	0.6	0	0	0
Ciprofloxacin	MIC_{50}	16	16	2	16	2	2	2	2	2	2
	MIC_{90}	16	16	16	16	16	16	16	16	16	16
	Resistance (%)	60.5	54.7	46.5	53.6	47.6	47.4	47.5	42.8	36.4	47.8
Chloramphenicol	MIC_{50}	8	8	8	8	8	8	8	8	8	8
	MIC_{90}	32	32	32	32	32	32	32	32	32	32
	Resistance (%)	22.8	19.4	16.9	21.4	24.0	15.4	15.5	18.8	23.4	35.1
Daptomycin	MIC_{50}	1	1	2	2	2	2	2	2	2	2
	MIC_{90}	2	2	2	2	2	2	4	2	2	2
	Resistance (%)	0	0.6	1.4	0	2.2	0.6	0	0	0	0
Erythromycin	MIC_{50}	32	32	64	64	16	16	64	32	2	8
	MIC_{90}	64	64	64	64	64	64	64	64	64	64
	Resistance (%)	76.5	74.7	72.8	64.3	59.4	53.7	64.1	63.0	45.5	51.5
Florfenicol	MIC_{50}	2	2	4	4	2	4	4	4	4	4
	MIC_{90}	2	4	4	32	32	16	32	32	32	32
	Resistance (%)	6.2	6.5	8.5	17.3	14.8	10.9	11.6	16.7	22.7	33.6
Gentamicin	MIC_{50}	128	128	128	128	128	128	128	128	128	128
	MIC_{90}	2048	1024	2048	2048	128	128	128	1024	128	256
	Resistance (%)	14.8	11.8	15.5	11.7	8.3	4.6	6.1	10.9	9.7	9.7
Kanamycin	MIC_{50}	128	128	128	128	256	128	256	128	128	128
	MIC_{90}	2048	2048	2048	2048	2048	2048	2048	2048	2048	2048
	Resistance (%)	23.5	24.7	26.8	24.0	21.4	12.0	14.4	23.2	21.4	21.6
Linezolid	MIC_{50}	1	1	2	2	1	2	2	2	2	2
	MIC_{90}	1	2	2	4	2	2	2	4	2	4
	Resistance (%)	0	0	0.5	3.6	0.4	1.1	0.6	2.2	1.3	2.2
Salinomycin	MIC_{50}	2	2	2	2	2	2	2	2	2	2
	MIC_{90}	4	4	4	4	4	4	4	4	2	2

	Resistance (%)	0	0.6	2.3	3.1	6.1	4.6	7.7	0.7	0	0
Streptomycin	MIC_{50}	1024	128	128	128	128	128	128	128	128	128
	MIC_{90}	2048	2048	2048	2048	2048	2048	2048	2048	2048	2048
	Resistance (%)	31.5	30.6	31.9	33.2	31.4	30.9	35.9	27.5	32.5	32.1
Tetracycline	MIC_{50}	128	128	128	128	128	128	128	128	64	64
	MIC_{90}	128	128	128	128	128	128	128	128	128	128
	Resistance (%)	79.0	85.3	78.4	77.0	80.8	76.0	77.9	71.0	66.2	61.9
Tigecycline	MIC_{50}	0.125	0.125	0.125	0.125	0.125	0.125	0.25	0.25	0.25	0.25
	MIC_{90}	0.25	0.125	0.25	0.25	0.25	0.5	0.5	0.5	0.25	0.25
	Resistance (%)	0	1.2	1.4	3.6	2.6	12.0	37.6	24.6	21.4	8.2
Tylosin	MIC_{50}	64	64	64	64	2	64	64	64	2	32
	MIC_{90}	64	64	64	64	64	64	64	64	64	64
	Resistance (%)	81.5	78.2	72.8	63.8	59.4	53.1	63.5	62.3	46.1	51.5
Vancomycin	MIC_{50}	2	2	2	2	2	2	2	2	2	2
	MIC_{90}	2	2	4	2	4	4	2	2	2	2
	Resistance (%)	0	0	0	0	0	0	0	0	0	0

MIC_{50} and MIC_{90} are the concentrations (μg/mL) at which 50% and 90% of the isolates were inhibited, respectively.

Ta**ble S6.** Antimicrobial resistance rate in *E. faecium* (n=572) isolated from cattle between 2010 and 2019 in Korea.

Antimicrobials	**2010 (n=25)**	**2011 (n=72)**	**2012 (n=53)**	**2013 (n=46)**	**2014 (n=73)**	**2015 (n=51)**	**2016 (n=57)**	**2017 (n=60)**	**2018 (n=78)**	**2019 (n=57)**	**Total (n=572)**	**P-value**
Ampicillin	0 (0)	0 (0)	0 (0)	2.2 (1)	0 (0)	0 (0)	0 (0)	0 (0)	0 (0)	1.8 (1)	0.3 (2)	0.5649
Chloramphenicol	8.0 (2)	1.4 (1)	0 (0)	2.2 (1)	2.7 (2)	5.9 (3)	1.8 (1)	0 (0)	1.3 (1)	3.5 (2)	2.3 (13)	0.4287
Ciprofloxacin	20.0 (5)	9.7 (7)	15.1 (8)	17.4 (8)	16.4 (12)	25.5 (13)	43.9 (25)	28.3 (17)	21.8 (17)	35.1 (20)	23.1 (132)	0.0329
Daptomycin	0 (0)	2.8 (2)	1.9 (1)	6.5 (3)	2.7 (2)	3.9 (2)	19.3 (11)	1.7 (1)	5.1 (4)	10.5 (6)	5.6 (32)	0.1596
Erythromycin	40.0(10)	30.6 (22)	13.2 (7)	6.5 (3)	6.8 (5)	9.8 (5)	8.8 (5)	30.0 (18)	34.6 (27)	21.1 (12)	19.9 (114)	0.8488
Florfenicol	8.0 (2)	2.8 (2)	0 (0)	0 (0)	2.7 (2)	0 (0)	0 (0)	1.7 (1)	1.3 (1)	1.8 (1)	1.6 (9)	0.1859
Gentamicin	4.0 (1)	0 (0)	0 (0)	0 (0)	0 (0)	0 (0)	0 (0)	0 (0)	0 (0)	0 (0)	0.2 (1)	0.1215
Kanamycin	12.0 (3)	26.4 (19)	1.9 (1)	8.7 (4)	2.7 (2)	3.9 (2)	0 (0)	3.3 (2)	1.3 (1)	3.5 (2)	6.3 (36)	0.0522
Linezolid	0 (0)	0 (0)	0 (0)	0 (0)	1.4 (1)	0 (0)	1.8 (1)	0 (0)	0 (0)	0 (0)	0.3 (2)	0.767
Quinupristin/dalfopristin	16.0 (4)	1.4 (1)	17.0 (9)	19.6 (9)	8.2 (6)	15.7 (8)	0 (0)	1.7 (1)	6.4 (5)	12.3 (7)	8.7 (50)	0.3691
Salinomycin	0 (0)	0 (0)	1.9 (1)	0 (0)	0 (0)	0 (0)	0 (0)	0 (0)	0 (0)	0 (0)	0.2 (1)	0.4161
Streptomycin	32.0 (8)	11.1 (8)	5.7 (3)	6.5 (3)	8.2 (6)	9.8 (5)	1.8 (1)	1.7 (1)	1.3 (1)	5.3 (3)	6.8 (39)	0.0249
Tetracycline	64.0(16)	47.2 (34)	30.2 (16)	15.2 (7)	26.0 (19)	21.6 (11)	35.1 (20)	16.7 (10)	9.0 (7)	14.0 (8)	25.9 (148)	0.0071
Tigecycline	4.0 (1)	1.4 (1)	1.9 (1)	0 (0)	1.4 (1)	7.8 (4)	10.5 (6)	11.7 (7)	15.4 (12)	10.5 (6)	6.8 (39)	0.0031
Tylosin	32.0 (8)	8.3 (6)	3.8 (2)	4.3 (2)	4.1 (3)	2.0 (1)	1.8 (1)	3.3 (2)	1.3 (1)	3.5 (2)	4.9 (28)	0.0489
Vancomycin	0 (0)	0 (0)	0 (0)	0 (0)	0 (0)	0 (0)	0 (0)	0 (0)	0 (0)	0(0)	0 (0)	-
MDR	32.0 (8)	12.5 (9)	7.5 (4)	8.7 (4)	5.5 (4)	11.8 (6)	10.5 (6)	3.3 (2)	1.3 (1)	8.8 (5)	8.6 (49)	0.0448

MDR, multidrug resistance; $P < 0.05$ was considered significant change in antibiotic resistance trend

Table S7. Antimicrobial resistance rate in *E. faecium* (n=1385) isolated from pigs between 2010 and 2019 in Korea.

Antimicrobials	2010 (n=60)	2011 (n=163)	2012 (n=148)	2013 (n=127)	2014 (n=144)	2015 (n=142)	2016 (n=152)	2017 (n=139)	2018 (n=197)	2019 (n=113)	Total (n=1385)	P-value
Ampicillin	0 (0)	0 (0)	0 (0)	0.8 (1)	0 (0)	0 (0)	0 (0)	0 (0)	0 (0)	0 (0)	0.1 (1)	0.6305
Chloramphenicol	5.0 (3)	2.5 (4)	5.4 (8)	3.9 (5)	11.1 (16)	12.0 (17)	6.6 (10)	7.2 (10)	7.1 (14)	7.1 (8)	6.9 (95)	0.2141
Ciprofloxacin	23.3 (14)	6.7 (11)	12.2 (18)	11.0 (14)	11.8 (17)	9.2 (13)	29.6 (45)	12.9 (18)	12.2 (24)	13.3 (15)	13.6 (189)	0.9697
Daptomycin	0 (0)	0 (0)	8.1 (12)	3.1 (4)	2.8 (4)	7.7 (11)	25.7 (39)	1.4 (2)	5.6 (11)	10.6 (12)	6.9 (95)	0.2304
Erythromycin	33.3 (20)	25.8 (42)	16.9 (25)	12.6 (16)	15.3 (22)	34.5 (49)	43.4 (66)	38.8 (54)	48.2 (95)	41.6 (47)	31.5 (436)	0.0402
Florfenicol	10.0 (6)	4.9 (8)	8.1 (12)	3.9 (5)	8.3 (12)	13.4 (19)	9.2 (14)	10.8 (15)	8.6 (17)	7.1 (8)	8.4 (116)	0.5254
Gentamicin	0 (0)	1.2 (2)	0.7 (1)	1.6 (2)	1.4 (2)	0 (0)	1.3 (2)	0 (0)	0 (0)	0.9 (1)	0.7 (10)	0.639
Kanamycin	21.7 (13)	10.4 (17)	7.4 (11)	9.4 (12)	8.3 (12)	7.7 (11)	11.8 (18)	6.5 (9)	8.6 (17)	10.6 (12)	9.5 (132)	0.1739
Linezolid	0 (0)	0 (0)	0 (0)	0 (0)	0 (0)	0 (0)	0 (0)	0 (0)	0 (0)	0 (0)	0 (0)	
Quinupristin/dalfopristin	8.3 (5)	3.1 (5)	25.0(37)	11.8 (15)	11.1 (16)	17.6 (25)	9.2 (14)	5.0 (7)	10.7 (21)	17.7 (20)	11.9 (165)	0.7784
Salinomycin	0 (0)	0 (0)	0.7 (1)	0.8 (1)	0.7 (1)	0 (0)	0.7 (1)	0 (0)	0.5 (1)	0 (0)	0.4 (5)	0.8905
Streptomycin	28.3 (17)	7.4 (12)	12.8 (19)	11.8 (15)	11.1 (16)	14.8 (21)	12.5 (19)	12.2 (17)	10.7 (21)	13.3 (15)	12.4 (172)	0.3063
Tetracycline	36.7 (22)	19.0 (31)	23.0 (34)	15.0 (19)	19.4 (28)	21.1 (30)	27.0 (41)	17.3 (24)	16.2 (32)	15.9 (18)	20.1 (279)	0.0998
Tigecycline	10.0 (6)	0.6 (1)	2.0 (3)	3.1 (4)	0.7 (1)	2.8 (4)	7.2 (11)	5.8 (8)	17.3 (34)	8.0 (9)	5.8 (81)	0.1708
Tylosin	23.3 (14)	7.4 (12)	12.8 (19)	11.0 (14)	12.5 (18)	16.2 (23)	14.5 (22)	13.7 (19)	17.3 (34)	14.2 (16)	13.8 (191)	0.9416
Vancomycin	0 (0)	0 (0)	0 (0)	0 (0)	0 (0)	0 (0)	0 (0)	0 (0)	0 (0)	0 (0)	0 (0)	
MDR	25.0 (15)	7.4 (12)	14.2 (21)	11.0 (14)	16.0 (23)	16.9 (24)	23.0 (35)	15.8 (22)	16.8 (33)	15.0 (17)	15.6 (216)	0.8381

MDR, multidrug resistance; $P < 0.05$ was considered significant change in antibiotic resistance trend.

Table S8. Antimicrobial resistance rate in *E. faecium* (n=1403) isolated from chickens between 2010 and 2019 in Korea.

Antimicrobials	2010 (n=77)	2011 (n=87)	2012 (n=168)	2013 (n=134)	2014 (n=240)	2015 (n=166)	2016 (n=162)	2017 (n=90)	2018 (n=168)	2019 (n=111)	Total (n=1403)	P-value
Ampicillin	13.0 (10)	12.6 (11)	9.5 (16)	10.4 (14)	8.3 (20)	12.7 (21)	4.3 (7)	12.2 (11)	13.7 (23)	24.3 (27)	11.4 (160)	0.2664
Chloramphenicol	22.1 (17)	19.5 (17)	16.1 (27)	15.7 (21)	19.2 (46)	14.5 (24)	14.8 (24)	12.2 (11)	26.2 (44)	29.7 (33)	18.8 (264)	0.4147
Ciprofloxacin	74.0 (57)	75.9 (66)	66.7 (112)	70.9 (95)	68.3 (164)	78.9 (131)	65.4 (106)	62.2 (56)	56.5 (95)	64.0 (71)	67.9 (953)	0.0283
Daptomycin	0 (0)	1.1 (1)	13.1 (22)	8.2 (11)	9.6 (23)	11.4 (19)	19.8 (32)	6.7 (6)	11.9 (20)	19.8 (22)	11.1 (156)	0.0212
Erythromycin	84.4 (65)	74.7 (65)	55.4 (93)	67.2 (90)	59.6 (143)	57.8 (96)	53.7 (87)	50.0(45)	45.2 (76)	67.6 (75)	59.5 (835)	0.0392
Florfenicol	24.7 (19)	21.8 (19)	14.9 (25)	14.2 (19)	18.8 (45)	15.1 (25)	13.0 (21)	12.2 (11)	26.2 (44)	30.6 (34)	18.7 (262)	0.6146
Gentamicin	10.4 (8)	1.1 (1)	3.6 (6)	0 (0)	1.7 (4)	2.4 (4)	1.9 (3)	2.2 (2)	0 (0)	4.5 (5)	2.4 (33)	0.2893
Kanamycin	20.8 (16)	3.4 (3)	12.5 (21)	9.7 (13)	11.7 (28)	11.4 (19)	6.2 (10)	8.9 (8)	7.7 (13)	9.9 (11)	10.1 (142)	0.2723
Linezolid	3.9 (3)	0 (0)	1.2 (2)	1.5 (2)	7.1 (17)	6.6 (11)	6.8 (11)	3.3 (3)	6.5 (11)	11.7 (13)	5.2 (73)	0.0158
Quinupristin/dalfopristin	39.0 (30)	26.4 (23)	44.0 (74)	34.3 (46)	29.2 (70)	34.3 (57)	24.1 (39)	6.7 (6)	14.9 (25)	20.7 (23)	28.0 (393)	0.014
Salinomycin	2.6 (2)	6.9 (6)	11.3 (19)	3.7 (5)	24.6 (59)	17.5 (29)	13.0(21)	2.2 (2)	3.0 (5)	5.4 (6)	11.0 (154)	0.8571
Streptomycin	50.6 (39)	46.0 (40)	41.1 (69)	44.0 (59)	30.4 (73)	38.0 (63)	16.7 (27)	23.3 (21)	13.1 (22)	17.1 (19)	30.8 (432)	0.0001
Tetracycline	87.0 (67)	82.8 (72)	70.8 (119)	77.6 (104)	62.9 (151)	63.9 (106)	48.8 (79)	52.2 (47)	42.9 (72)	44.1 (49)	61.7 (866)	0
Tigecycline	3.9 (3)	1.1 (1)	1.8 (3)	3.0 (4)	4.6 (11)	9.6 (16)	9.3 (15)	10.0 (9)	5.4 (9)	5.4 (6)	5.5 (77)	0.0581
Tylosin	84.4 (65)	74.7 (65)	51.8 (87)	64.9 (87)	58.8 (141)	51.2 (85)	38.3 (62)	41.1 (37)	35.7 (60)	48.6 (54)	53.0(743)	0.0019
Vancomycin	0 (0)	0 (0)	0 (0)	0 (0)	0 (0)	0 (0)	0 (0)	0 (0)	0 (0)	0 (0)	0 (0)	
MDR	84.4 (65)	77.0 (67)	62.5 (105)	70.9 (95)	65.4 (157)	65.7 (109)	50.0 (81)	46.7 (42)	39.9 (67)	59.5 (66)	60.9 (854)	0.0027

MDR, multidrug resistance; $P < 0.05$ was considered significant change in antibiotic resistance trend.

Table S9. Antimicrobial resistance rate in *E. faecalis* (n=910) isolated from cattle between 2010 and 2019 in Korea.

Antimicrobials	2010 (n=61)	2011 (n=152)	2012 (n=80)	2013 (n=85)	2014 (n=99)	2015 (n=102)	2016 (n=95)	2017 (n=76)	2018 (n=80)	2019 (n=80)	Total (n=910)	P-value
Ampicillin	0 (0)	0 (0)	0 (0)	0 (0)	0 (0)	0 (0)	0 (0)	0 (0)	0 (0)	0 (0)	0 (0)	
Chloramphenicol	16.4 (10)	15.1 (23)	16.3 (13)	3.5 (3)	11.1 (11)	19.6 (20)	10.5 (10)	7.9 (6)	6.3 (5)	11.3 (9)	12.1 (110)	0.2064
Ciprofloxacin	0 (0)	9.9 (15)	8.8 (7)	9.4 (8)	8.1 (8)	12.7 (13)	13.7 (13)	3.9 (3)	3.8 (3)	2.5 (2)	7.9 (72)	0.7617
Daptomycin	0 (0)	0.7 (1)	1.3 (1)	0 (0)	2.0 (2)	2.0 (2)	3.2 (3)	1.3 (1)	1.3 (1)	1.3 (1)	1.3 (12)	0.1626
Erythromycin	24.6 (15)	32.2 (49)	23.8 (19)	17.6 (15)	13.1 (13)	19.6 (20)	23.2 (22)	11.8 (9)	16.3 (13)	13.8 (11)	20.4 (186)	0.0224
Florfenicol	0 (0)	0.7 (1)	6.3 (5)	3.5 (3)	3.0 (3)	8.8 (9)	8.4 (8)	5.3 (4)	3.8 (3)	6.3 (5)	4.5 (41)	0.0795
Gentamicin	13.1 (8)	8.6 (13)	8.8 (7)	7.1 (6)	9.1 (9)	5.9 (6)	7.4 (7)	2.6 (2)	5.0 (4)	7.5 (6)	7.5 (68)	
Kanamycin	14.8 (9)	15.8 (24)	16.3 (13)	15.3 (13)	11.1 (11)	10.8 (11)	18.9 (18)	5.3 (4)	12.5 (10)	11.3 (9)	13.4 (122)	0.1646
Linezolid	0 (0)	0 (0)	0 (0)	0 (0)	0 (0)	2.0 (2)	0 (0)	0 (0)	0 (0)	0 (0)	0.2 (2)	0.8735
Salinomycin	0 (0)	0 (0)	0 (0)	0 (0)	0 (0)	0 (0)	0 (0)	0 (0)	0 (0)	0 (0)	0 (0)	
Streptomycin	23.0 (14)	19.1 (29)	27.5 (22)	22.4 (19)	24.2 (24)	18.6 (19)	21.1 (20)	11.8 (9)	10.0 (8)	8.8 (7)	18.8 (171)	0.0051
Tetracycline	54.1 (33)	52.0 (79)	58.8 (47)	43.5 (37)	46.5 (46)	42.2 (43)	35.8 (34)	27.6 (21)	22.5 (18)	27.5 (22)	41.8 (380)	0.0001
Tigecycline	0 (0)	0 (0)	3.8 (3)	1.2 (1)	0 (0)	7.8 (8)	20.0 (19)	22.4 (17)	12.5 (10)	12.5 (10)	7.5 (68)	0.0088
Tylosin	24.6 (15)	31.6 (48)	22.5 (18)	17.6 (15)	13.1 (13)	19.6 (20)	22.1 (21)	11.8 (9)	16.3 (13)	13.8 (11)	20.1 (183)	0.0197
Vancomycin	0 (0)	0 (0)	0 (0)	0 (0)	0 (0)	0 (0)	0 (0)	0 (0)	0 (0)	0 (0)	0 (0)	
MDR	21.3 (13)	24.3 (37)	28.8 (23)	17.6 (15)	16.2 (16)	25.5 (26)	20.0 (19)	11.8 (9)	12.5 (10)	13.8 (11)	19.7 (179)	0.0269

MDR, multidrug resistance; $P < 0.05$ was considered significant change in antibiotic resistance trend.

Table S10. Antimicrobial resistance rate in *E. faecalis* (n=1556) isolated from pigs between 2010 and 2019 in Korea.

Antimicrobials	2010 (n=88)	2011 (n=216)	2012 (n=145)	2013 (n=166)	2014 (n=164)	2015 (n=173)	2016 (n=200)	2017 (n=155)	2018 (n=143)	2019 (n=106)	Total (n=1556)	P-value
Ampicillin	0 (0)	0.5 (1)	0 (0)	0 (0)	0 (0)	0 (0)	0 (0)	0 (0)	0 (0)	0 (0)	0.1 (1)	0.2441
Chloramphenicol	45.5 (40)	48.6 (105)	44.1 (64)	48.8 (81)	46.3 (76)	55.5 (96)	41.0 (82)	61.3 (95)	55.2 (79)	56.6 (60)	50.0 (778)	0.0586
Ciprofloxacin	9.1 (8)	13.4 (29)	9.7 (14)	10.8 (18)	12.8 (21)	16.8 (29)	17.5 (35)	15.5 (24)	18.9 (27)	7.5 (8)	13.7 (213)	0.2981
Daptomycin	0 (0)	0.5 (1)	0 (0)	1.2 (2)	1.2 (2)	1.7 (3)	0.5 (1)	0 (0)	0 (0)	0.9 (1)	0.6 (10)	0.7842
Erythromycin	64.8 (57)	67.1 (145)	59.3 (86)	65.1 (108)	60.4 (99)	70.5 (122)	63.5 (127)	75.5 (117)	74.8 (107)	71.7 (76)	67.1 (1044)	0.0404
Florfenicol	23.9 (21)	31.5 (68)	40.7 (59)	42.8 (71)	53.7 (88)	52.6 (91)	43.5 (87)	55.5 (86)	57.3 (82)	55.7 (59)	45.8 (712)	0.0009
Gentamicin	22.7 (20)	18.1 (39)	20.0 (29)	17.5 (29)	14.0 (23)	23.1 (40)	12.5 (25)	16.8 (26)	24.5 (35)	26.4 (28)	18.9 (294)	0.5306
Kanamycin	47.7 (42)	48.6 (105)	44.8 (65)	41.0 (68)	39.0 (64)	47.4 (82)	34.5 (69)	44.5 (69)	52.4 (75)	48.1 (51)	44.3 (690)	0.8663
Linezolid	0 (0)	0.5 (1)	0.7 (1)	0.6 (1)	0 (0)	2.3 (4)	1.0 (2)	1.9 (3)	2.8 (4)	5.7 (6)	1.4 (22)	0.0044
Salinomycin	0 (0)	0 (0)	0 (0)	0 (0)	0 (0)	0 (0)	0 (0)	0 (0)	0 (0)	0 (0)	0 (0)	
Streptomycin	51.1 (45)	45.4 (98)	51.0 (74)	45.8 (76)	48.8 (80)	60.1 (104)	46.5 (93)	52.3 (81)	59.4 (85)	52.8 (56)	50.9 (792)	0.167
Tetracycline	75.0 (66)	80.6 (174)	80.7 (117)	77.7 (129)	76.2 (125)	83.2 (144)	73.0 (146)	81.9 (127)	79.7 (114)	72.6 (77)	78.3 (1219)	0.6947
Tigecycline	0 (0)	0.9 (2)	4.1 (6)	3.0 (5)	1.8 (3)	9.8 (17)	33.5 (67)	27.1 (42)	16.1 (23)	13.2 (14)	11.5 (179)	0.043
Tylosin	64.8 (57)	67.1 (145)	59.3 (86)	65.1 (108)	59.8 (98)	70.5 (122)	62.0 (124)	73.5 (114)	74.1 (106)	71.7 (76)	66.6 (1036)	0.0593
Vancomycin	0 (0)	0 (0)	0 (0)	0 (0)	0 (0)	0 (0)	0 (0)	0 (0)	0 (0)	0 (0)	0 (0)	
MDR	56.8 (50)	59.7 (129)	57.2 (83)	59.0 (98)	59.8 (98)	74.0 (128)	59.5 (119)	72.3 (112)	69.9 (100)	68.9 (73)	63.6 (990)	0.0141

MDR, multidrug resistancel; $P < 0.05$ was considered significant change in antibiotic resistance trend.

Table S11. Antimicrobial resistance rate in *E. faecalis* (n=1752) isolated from chickens between 2010 and 2019 in Korea.

**Antimicrobials**	**2010 (n=162)**	**2011 (n=170)**	**2012 (n=213)**	**2013 (n=196)**	**2014 (n=229)**	**2015 (n=175)**	**2016 (n=181)**	**2017 (n=138)**	**2018 (n=154)**	**2019 (n=134)**	**Total (n=1752)**	**P-value**
Ampicillin	0 (0)	0 (0)	0 (0)	0 (0)	0 (0)	0.6 (1)	0.6 (1)	0 (0)	0 (0)	0 (0)	0.1 (2)	0.6305
Chloramphenicol	22.8 (37)	19.4 (33)	16.9 (36)	21.4 (42)	24.0 (55)	15.4 (27)	15.5 (28)	18.8 (26)	23.4 (36)	35.1 (47)	20.9 (367)	0.2708
Ciprofloxacin	60.5 (98)	54.7 (93)	46.5 (99)	53.6 (105)	47.6 (109)	47.4 (83)	47.5 (86)	42.8 (59)	36.4 (56)	47.8 (64)	48.6 (852)	0.0085
Daptomycin	0 (0)	0.6 (1)	1.4 (3)	0 (0)	2.2 (5)	0.6 (1)	0 (0)	0 (0)	0 (0)	0 (0)	0.6 (10)	0.3858
Erythromycin	76.5 (124)	74.7 (127)	72.8 (155)	64.3 (126)	59.4 (136)	53.7 (94)	64.1 (116)	63.0 (87)	45.5 (70)	51.5 (69)	63.0 (1104)	0.0013
Florfenicol	6.2 (10)	6.5 (11)	8.5 (18)	17.3 (34)	14.8 (34)	10.9 (19)	11.6 (21)	16.7 (23)	22.7 (35)	33.6 (45)	14.3 (250)	0.0029
Gentamicin	14.8 (24)	11.8 (20)	15.5 (33)	11.7 (23)	8.3 (19)	4.6 (8)	6.1 (11)	10.9 (15)	9.7 (15)	9.7 (13)	10.3 (181)	0.0961
Kanamycin	23.5 (38)	24.7 (42)	26.8 (57)	24.0 (47)	21.4 (49)	12.0 (21)	14.4 (26)	23.2 (32)	21.4 (33)	21.6 (29)	21.3 (374)	0.2764
Linezolid	0 (0)	0 (0)	0.5 (1)	3.6 (7)	0.4 (1)	1.1 (2)	0.6 (1)	2.2 (3)	1.3 (2)	2.2 (3)	1.1 (20)	0.1772
Salinomycin	0 (0)	0.6 (1)	2.3 (5)	3.1 (6)	6.1 (14)	4.6 (8)	7.7 (14)	0.7 (1)	0 (0)	0 (0)	2.8 (49)	0.9986
Streptomycin	31.5 (51)	30.6 (52)	31.9 (68)	33.2 (65)	31.4 (72)	30.9 (54)	35.9 (65)	27.5 (38)	32.5 (50)	32.1 (43)	31.8 (558)	0.9203
Tetracycline	79.0 (128)	85.3 (145)	78.4 (167)	77.0 (151)	80.8 (185)	76.0 (133)	77.9 (141)	71.0 (98)	66.2 (102)	61.9 (83)	76.1 (1333)	0.0017
Tigecycline	0 (0)	1.2 (2)	1.4 (3)	3.6 (7)	2.6 (6)	12.0 (21)	37.6 (68)	24.6 (34)	21.4 (33)	8.2 (11)	10.6 (185)	0.0457
Tylosin	81.5 (132)	78.2 (133)	72.8 (155)	63.8 (125)	59.4 (136)	53.1 (93)	63.5 (115)	62.3 (86)	46.1 (71)	51.5 (69)	63.6 (1115)	0.0007
Vancomycin	0 (0)	0 (0)	0 (0)	0 (0)	0 (0)	0 (0)	0 (0)	0 (0)	0 (0)	0 (0)	0 (0)	-
MDR	72.8 (118)	68.2 (116)	62.4 (133)	60.2 (118)	58.1 (133)	54.3 (95)	62.4 (113)	58.0 (80)	46.8 (72)	50.7 (68)	59.7 (1046)	0.001

MDR, multidrug resistance; $P < 0.05$ was considered significant change in antibiotic resistance trend.
